# OTUB1 augments hypoxia signaling via its non-canonical ubiquitination inhibition of HIF-1α during hypoxia adaptation

**DOI:** 10.1038/s41419-022-05008-z

**Published:** 2022-06-22

**Authors:** Xing Liu, Hongyan Deng, Jinhua Tang, Zixuan Wang, Chunchun Zhu, Xiaolian Cai, Fangjing Rong, Xiaoyun Chen, Xueyi Sun, Shuke Jia, Gang Ouyang, Wenhua Li, Wuhan Xiao

**Affiliations:** 1 State Key Laboratory of Freshwater Ecology and Biotechnology, Institute of Hydrobiology, Chinese Academy of Sciences, Wuhan, PR China; 2 University of Chinese Academy of Sciences, Beijing, 100049 PR China; 3 The Innovation of Seed Design, Chinese Academy of Sciences, Wuhan, 430072 PR China; 4 Hubei Hongshan Laboratory, Wuhan, 430070 PR China; 5 College of Life Science, Wuhan University, Wuhan, 430072 PR China; 6 Hubei Key Laboratory of Cell Homeostasis, College of Life Sciences, Wuhan University, Wuhan, 430072 PR China

**Keywords:** Deubiquitylating enzymes, Transcription factors

## Abstract

As a main regulator of cellular responses to hypoxia, the protein stability of hypoxia-inducible factor (HIF)-1α is strictly controlled by oxygen tension dependent of PHDs-catalyzed protein hydroxylation and pVHL complex-mediated proteasomal degradation. Whether HIF-1α protein stability as well as its activity can be further regulated under hypoxia is not well understood. In this study, we found that OTUB1 augments hypoxia signaling independent of PHDs/VHL and FIH. OTUB1 binds to HIF-1α and depletion of OTUB1 reduces endogenous HIF-1α protein under hypoxia. In addition, OTUB1 inhibits K48-linked polyubiquitination of HIF-1α via its non-canonical inhibition of ubiquitination activity. Furthermore, OTUB1 promotes hypoxia-induced glycolytic reprogramming for cellular metabolic adaptation. These findings define a novel regulation of HIF-1α under hypoxia and demonstrate that OTUB1-mediated HIF-1α stabilization positively regulates HIF-1α transcriptional activity and benefits cellular hypoxia adaptation.

## Introduction

Hypoxia signaling regulation is the most important cellular process for adapting to physiological oxygen variations, and its dysregulation leads to various pathophysiological dysfunction [1–4]. The master molecules of the hypoxia signaling are the hypoxia-inducible factors (HIF-1 and HIF-2), which widely controls oxygen responsive genes involved in energy metabolism, angiogenesis, apoptosis, etc. [1, 2, 5, 6]. The α subunit of HIF-1 or HIF-2 is rapidly degraded under normoxia; conversely, this subunit is stabilized when O_2_-dependent degradation is inhibited under hypoxia [7, 8]. The stability and activity of the α subunit of HIF are regulated by its post-translational modifications, including hydroxylation, acetylation, SUMOylation, phosphorylation, methylation and ubiquitination [9–20]. Particularly, the prolyl hydroxylases (PHD1, PHD2, PHD3 or called EglN2, EglN1, EglN3, respectively) hydroxylate a specific residue of the HIF-α subunit (Pro402 and Pro564 on HIF-1α, Pro405 and Pro531 on HIF-2α), to trigger a ubiquitination reaction by E3 ubiquitin ligase Von Hippel-Lindau protein (pVHL) and proteasome-mediated degradation, which represents a canonical hypoxia signaling pathway [21–24]. While ubiquitination is a powerful HIF regulatory modification, deubiquitination of HIF and its impact on hypoxia signaling is also gotten much more attentions [25, 26].

DUBs are enzymes that can remove ubiquitin chains from proteins [27]. Based on sequences and structure, the DUBs can be divided into seven subgroups, including the ubiquitin-specific proteases (USPs), the ubiquitin carboxyl-terminal hydrolases (UCHs), the otubain/ovarian tumor-domain containing proteins (OTUs), the Machado-Joseph diseases domain superfamily (MJDs), the JAB1/MPN/MOV34 proteases (JAMMs), the monocyte chemotactic protein-induced proteins (MCPIPs), and the novel motif interacting with ubiquitin DUB family (MINDY) [25, 27]. The DUBs play a major role in protecting proteins from proteasomal degradation, thereby affecting various signaling pathway directly or indirectly [28, 29]. Even through it is relatively superficial in understanding the role of deubiquitination in the hypoxia signaling pathway compared to that of ubiquitination, to date, several DUBs, such as *USP7, USP8, USP19, USP20, USP28, USP37, UCHL1* and *OTUD7B*, have been identified to target HIF-α for deubiquitination, resulting in either the enhancement of HIF activity or the suppression of HIF activity [30–37].

OTUB1 (OUT domain-containing ubiquitin aldehyde-binding protein 1) was previously identified as a K48 linkage-specific deubiquitinating enzyme associated with the prevention of protein degradation [38]. OTUB1 has been reported to modulate multiple signaling pathways by deubiquitinating signaling molecules, such as *p100*, *UBE2F1, snail, DEPTOR, YB-1, SMAD2/3, c-IAP, p53, AKT, SOCS1, UBC13, PD-L1, Cyclin E1, MSH2, SLC7A11, TRAF3*, and *Nur77* [39–57]. Interestingly, the enzymatic activity of OTUB1 can be regulated by factor inhibiting HIF (FIH)-mediated hydroxylation in an oxygen-dependent manner [58, 59]. In fact, FIH is a well-characterized hydroxylase that catalyzes hydroxylation of asparagine residue within HIF-α subunits dependent on oxygen, resulting in the inhibition of HIF-dependent transcription under normoxia [60–64]. This phenomenon provoked us to investigate the impact of OTUB1 on hypoxia signaling.

In this study, we found that OTUB1 augments hypoxia signaling independent of PHDs/VHL and FIH. OTUB1 binds to HIF-1α and depletion of OTUB1 reduces endogenous HIF-1α protein under hypoxia. Furthermore, we found that OTUB1 inhibits K48-linked polyubiquitination of HIF-1α via its non-canonical inhibition of ubiquitination activity.

## Materials and methods

### Cell line and culture conditions

HEK293T and H1299 cells originally obtained from American Type Culture Collection (ATCC) were cultured in Dulbeccos’ modified Eagle medium (DMEM) (HyClone) with 10% fetal bovine serum (FBS). The cells were grown at 37 °C in a humidified incubator containing 5% CO_2_. The cells were cultured under hypoxic condition (1% O_2_, 5% CO_2_, and balanced with N_2_) by using the NBS Galaxy 48 R incubator.

### Antibodies and chemical reagents

Antibodies including anti-OTUB1 (#3783), anti-HIF-1α (#36169), anti-VHL (#68547), anti-FIH (#4426), anti-Ubiquitin (#3936), anti-K48-linkage Specific Polyubiquitin (#8081), and normal rabbit IgG (#2729) were purchased from Cell Signaling Technology. Anti-ACTB (#AC026) antibody was purchased from ABclonal. Anti-Flag (#F1804) antibody was purchased from Sigma. Anti-HA (#901515) antibody was purchased from Covance. Anti-Myc (#SC-40) antibody was purchased from Santa Cruz Biotechnology. CoCl_2_ (#C8661), Deferoxamine mesylate salt (DFX) (#D9533), DMOG (#D3695) and MG-132 (#474790) were purchased from Sigma. FG4592 (#S1007) was purchased from Selleck. The cells were treated with DMOG (1 mM) or FG4592 (up to 100 μM) for 6–8 h, and DMSO was used as a control.

### Quantitative real-time PCR assay

Total RNAs were extracted using RNAiso Plus (TaKaRa Bio., Beijing, China) following the protocol provided by the manufacturer. cDNAs were synthesized using the Revert Aid First Strand cDNA Synthesis Kit (Thermo Scientific, Waltham, MA, USA). MonAmp^TM^ SYBR^®^ Green qPCR Mix (high Rox) (Monad Bio., Shanghai, China) was used for quantitative RT–PCR assays (qPCR). The primers for quantitative RT- PCR assays are listed in Supplementary Table S1.

### Immunoprecipitation and Western blot

Co-immunoprecipitation and Western blot analysis were performed as described previously [20]. Anti-Flag antibody-conjugated agarose beads (#A2220), anti-HA antibody-conjugated agarose beads (#A2095) and anti-Myc antibody-conjugated agarose beads (#A7470) were purchased from Sigma. Protein G Sepharose (#17–0618–01) was purchased from GE HealthCare Company. The Fuji Film LAS4000 mini-luminescent image analyzer was used to photograph the blots. Image J software (National Institutes of Health) was used to quantify protein levels based on the band density obtained by Western blot analysis.

### CRISPR-Cas9 knockout cell lines

To generate H1299 or HEK293T knocked-out cell lines of indicated genes, sgRNA sequence were ligated into Lenti-CRISPRv2 plasmid and then co-transfected with viral packaging plasmids (psPAX2 and pMD2G) into HEK293T cells. Six hours after transfection, medium was changed, and viral supernatant was collected and filtered through 0.45-μm strainer. Targeted cells were infected by viral supernatant and selected by 1 μg/ml puromycin for 2 weeks. The sgRNA sequence targeting *VHL* was described as previously [65]. The sgRNA sequence targeting *OTUB1* is: GGTCCTGCTGAGCCATGA. The sgRNA sequence targeting *FIH* is: GGGTCGCTCTGACTCAGACG. The sgRNA sequence targeting *HIF-1β* is: GTCGCCGCTTAATAGCCCTC.

### Ubiquitination assay

Ubiquitination assays were followed the protocol described previously with some modifications [66]. Briefly, HEK293T cells were co-transfected with the plasmids expressing Myc-HIF-1α, His-ubiquitin or His-ubiquitin-K48 or His-ubiquitin-K48R, together with Flag-OTUB1 or Flag empty as a control for 24 h and then lysed by denatured buffer (6 M guanidine-HCl, 0.1 M Na_2_HPO_4_/NaH_2_PO_4_, 10 mM imidazole), followed by nickel bead purification and immunoblotting with the indicated antibodies.

For ubiquitination assay in *OTUB1*-deficient or wildtype H1299 cells (*OTUB1*^−/−^ or *OTUB1*^+/+^), the cells were treated with or without MG-132 (20 μM) for 6~8 h, then collected, and lysed with the lysis buffer (100 μl). The supernatants were denatured at 95 °C for 5 min in the presence of 1% SDS. The denatured lysates were diluted with lysis buffer to reduce the concentration of SDS (less than 0.1%). Immunoprecipitation (denature-IP) was conducted using anti-HIF-1α antibody and then subjected to immunoblotting with anti-Ubiquitin or anti-K48-linkage specific polyubiquitin antibody.

### Cell proliferation assay

*OTUB1*-deficient or wildtype H1299 cells (*OTUB1*^−/−^ or *OTUB1*^+/+^) seeded in 96-well plates at 500 cells per well were cultured for indicated days, and CCK-8 assay was employed to determinate the cell growth rate according to the manufacturer’s instructions.

### Colony formation assays

*OTUB1*-deficient or wildtype H1299 cells (*OTUB1*^−/−^ or *OTUB1*^+/+^) seeded in 6-well plates at 2 × 10^3^ cells per well were cultured under hypoxia (1% O_2_). After 7 days, the colonies were fixed by methanol, stained with crystal violet (0.5% in methanol) and washed with PBS, and then photographed. Colonies of a suitable size were counted based on the images in each well.

### Mitochondrial stress test and glycolytic rate test assays

The oxygen consumption rate (OCR) under mitochondrial stress test assay and the proton efflux rate (PER) under glycolytic rate test assay were performed using the Seahorse XFe24 Extracellular Flux Analyzer (Agilent Technologies, Santa Clara, CA, USA). Mitochondrial stress and glycolytic rate test assays were performed using XF Cell Mito Stress Test kit (Agilent Technologies, #103015–100) and XF Glycolytic rate Assay Kit (Agilent Technologies, #103344–100), respectively. The assays were performed according to the manufacturer’s instructions. The H1299 cells (4 × 10^4^ cells/well) were cultured in XF24 cell culture microplate (Agilent Technologies, #102340–100). For mitochondrial stress test assay, oligomycin (1.5 μM), carbonyl cyanide-4-(trifluoromethoxy) phenylhydrazone (FCCP, 2 μM) and antimycin A and rotenone mixture (0.5 μM) were added to cell culture plate for determining mitochondrial respiration including basal respiration, maximal respiration and spare respiratory capacity. For glycolytic rate test assay, antimycin A and rotenone mixture (0.5 μM) and 2-deoxy-D-glucose (50 mM) were added to cell culture plate for determining glycolytic flux including basal glycolysis and compensatory glycolysis.

### Glucose uptake assay

Glucose uptake was analyzed directly using the fluorescent glucose analog 2-NBDG. *OTUB1*-deficient or wildtype H1299 cells (*OTUB1*^−/−^ or *OTUB1*^+/+^) were incubated in glucose-free DMEM medium overnight and then cultured under hypoxia for 6 h. 50 μM 2-NBDG was added into the medium and then the cells were incubated for 1 h at 37 °C in dark, and the amount of 2-NBDG taken up by cells was detected by fluorescence microscopy or assessed by flow cytometry analysis (FACS).

### Statistical analysis

GraphPad Prism software (7.0) was used for all statistical analysis. Results with error bars express mean ± SD. Statistical analysis was performed as indicated in figure legends. A *P* value less than 0.05 was considered significant. Statistical significance is represented as follows: *p < 0.05, **p < 0.01, ***p < 0.001, ****p < 0.0001.

## Results

### OTUB1 augments hypoxia signaling

The oxygen-dependent regulation of OTUB1 by FIH provoked us to determinate whether OTUB1 has impact on hypoxia signaling [58, 59]. Initially, we checked the effect of hypoxia on the formation of FIH and OTUB1 heterodimers. In fact, a heterotrimeric complex is indeed formed, which is consisted of FIH and OTUB1 (Fig. S1A). In addition, the formation of this complex is dependent on the hydroxylation of OTUB1 on N22 by FIH and sensitive to hypoxia (Fig. S1B, C), in agreement with the previous report [58, 59]. Furthermore, hypoxia could induce the expression of OTUB1 (Fig. S2A, B). These results implicated that OTUB1 may involve in hypoxia signaling.

**Fig. 1:**
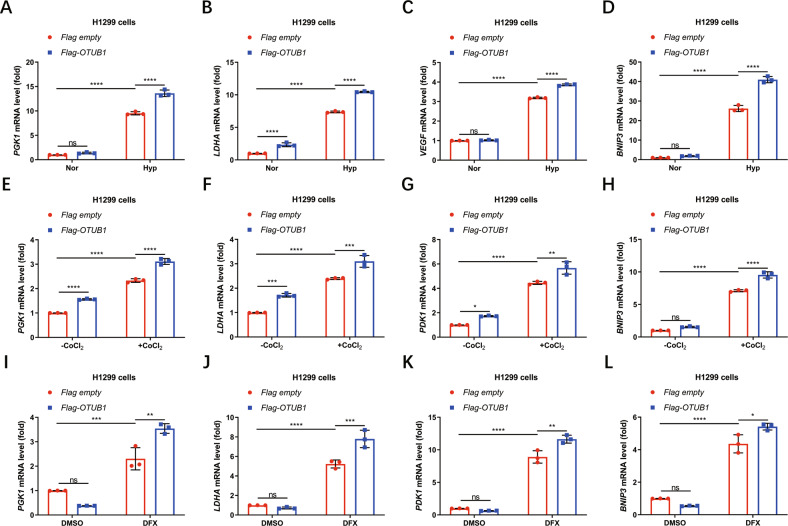
OTUB1 augments hypoxia signaling. **A**–**D** qPCR analysis of *PGK1* (**A**), *LDHA* (**B**), *VEGF* (**C**), and *BNIP3* (**D**) mRNA in H1299 cells transfected with or without Flag-OTUB1 and cultured under normoxia (21% O_2_) or hypoxia (1% O_2_) for 24 h. Flag empty vector was used as a control. **E**–**H** qPCR analysis of *PGK1* (**E**), *LDHA* (**F**), *PDK1* (**G**), and *BNIP3* (**H**) mRNA in H1299 cells transfected with or without Flag-OTUB1 and treated with or without CoCl_2_ (200 μM) for 8 h. Flag empty vector was used as a control. **I**–**L** qPCR analysis of *PGK1* (**I**), *LDHA* (**J**), *PDK1* (**K**), and *BNIP3* (**L**) mRNA in H1299 cells transfected with or without Flag-OTUB1 and treated with DFX (150 μM) or DMSO as a control for 8 h. Flag empty vector was used as a control. Two-way ANOVA analysis; Data show mean ± SD; Tukey’s multiple comparisons test; ns, not significant, *Adjusted *p* < 0.05, **Adjusted *p* < 0.01, ***Adjusted *p* < 0.001, ****Adjusted *p* < 0.0001; Data from 3 independent experiments.

**Fig. 2:**
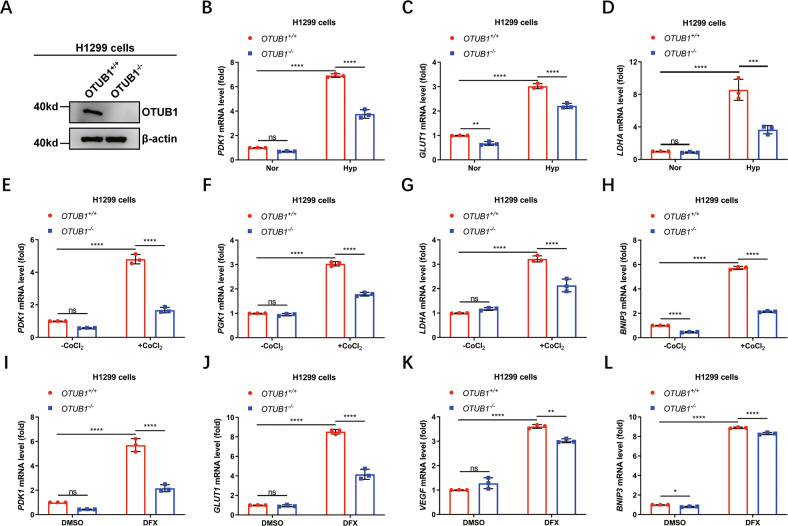
Loss of OTUB1 diminishes hypoxia signaling. **A** Western blot analysis of indicated proteins in *OTUB1*-deficient or wildtype H1299 cells (*OTUB1*^−/−^ or *OTUB1*^+/+^). **B**–**D** qPCR analysis of *PDK1* (**B**), *GLUT1* (**C**), and *LDHA* (**D**) mRNA in *OTUB1*-deficient or wildtype H1299 cells (*OTUB1*^−/−^ or *OTUB1*^+/+^) cultured under normoxia (21% O_2_) or hypoxia (1% O_2_) for 24 h. Two-way ANOVA analysis; Data show mean ± SD; Tukey’s multiple comparisons test; ns, not significant, *Adjusted *p* < 0.05, **Adjusted *p* < 0.01, ***Adjusted *p* < 0.001, ****Adjusted *p* < 0.0001; Data from 3 independent experiments. **E**–**H** qPCR analysis of *PDK1* (**E**), *PGK1* (**F**), *LDHA* (**G**) and *BNIP3* (**H**) mRNA in *OTUB1*-deficient or wildtype H1299 cells (*OTUB1*^−/−^ or *OTUB1*^+/+^) treated with or without CoCl_2_ (200 μM) for 8 h. Two-way ANOVA analysis; Data show mean ± SD; Tukey’s multiple comparisons test; ns, not significant, *Adjusted *p* < 0.05, **Adjusted *p* < 0.01, ***Adjusted *p* < 0.001, ****Adjusted *p* < 0.0001; Data from 3 independent experiments. **I**–**L** qPCR analysis of *PDK1* (**I**), *GLUT1* (**J**), *VEGF* (**K**), and *BNIP3* (**L**) mRNA in *OTUB1*-deficient or wildtype H1299 cells (*OTUB1*^−/−^ or *OTUB1*^+/+^) treated with DMSO or DFX (150 μM) for 8 h. Two-way ANOVA analysis; Data show mean ± SD; Tukey’s multiple comparisons test; ns, not significant, *Adjusted *p* < 0.05, **Adjusted *p* < 0.01, ***Adjusted *p* < 0.001, ****Adjusted *p* < 0.0001; Data from 3 independent experiments.

Subsequently, we checked the effect of OTUB1 on the expression of hypoxia responsive genes in cells after treated with hypoxia or hypoxia-mimic conditions [1, 20]. Overexpression of *OTUB1* in H1299 cells enhanced expression of *PGK1, LDHA, VEGF* and *BNIP3* under hypoxia (1% O_2_) as revealed by quantitative real-time PCR assays (qPCR) (Fig. 1A–D, S3F) [20, 67]. Similar results were obtained in HEK293T cells (Fig. S3A–E). Consistently, when the cells were treated with either CoCl_2_ or Deferoxamine mesylate salt (DFX) [68, 69], two hypoxia-mimic conditions, the increased expression of *PGK1, LDHA, PDK1* and *BNIP3* was also observed in *OTUB1*-overexpressed H1299 cells (Figs. 1E–L, S3F). Subsequently, we knocked out *OTUB1* in H1299 cells via CRISPR/Ca9 technique and examined hypoxia responsive gene expression (Fig. 2A). Under hypoxia (1% O_2_), expression of *PDK1, GLUT1* and *LDHA* in *OTUB1*^−/−^ H1299 cells was lower than that in *OTUB1*^+/+^ H1299 cells (Fig. 2B–D). In agreement, expression of *PDK1, PGK1, GLUT1, LDHA, BNIP3* or *VEGF* in *OTUB1*^−/−^ H1299 cells was lower than that in *OTUB1*^+/+^ H1299 cells when cells were treatment with either CoCl_2_ or DFX (Fig. 2E–L). However, reconstitution of *OTUB1* in *OTUB1*^−/−^ H1299 cells promoted expression of *PDK1, PGK1, GLUT1* and *LDHA* compared to reconstitution of empty vector control under hypoxia (1% O_2_) (Fig. S4A–D, H). These data suggest that OTUB1 enhances HIF transactivity under hypoxia.

**Fig. 3:**
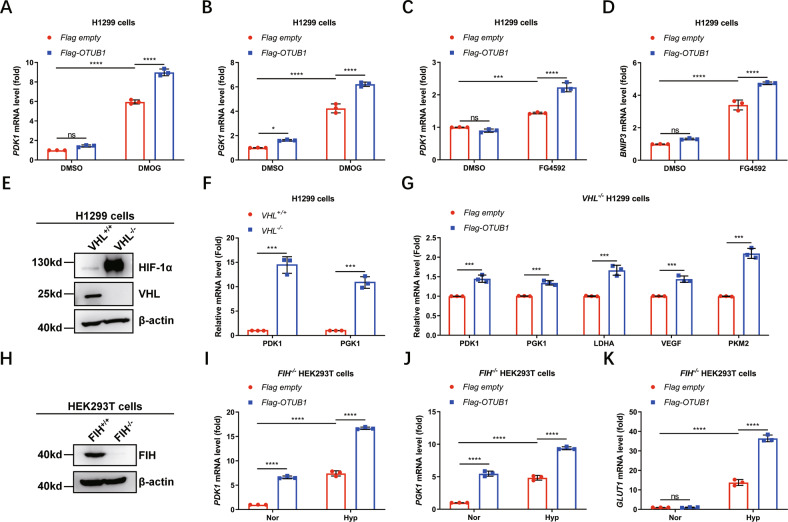
OTUB1 augments hypoxia signaling pathway independent of HIF-1α hydroxylation. **A**, **B** qPCR analysis of *PDK1* (**A**) and *PGK1* (**B**) mRNA in H1299 cells transfected with or without Flag-OTUB1 and cultured under normoxia (21% O_2_) for 24 h, followed by treatment with DMSO or DMOG (1 mM) for 8 h. Flag empty vector was used as a control. Two-way ANOVA analysis; Data show mean ± SD; Tukey’s multiple comparisons test; ns, not significant, *Adjusted *p* < 0.05, **Adjusted *p* < 0.01, ***Adjusted *p* < 0.001, ****Adjusted *p* < 0.0001; Data from 3 independent experiments. **C**, **D** qPCR analysis of *PDK1* (**C**) and *BNIP3* (**D**) mRNA in H1299 cells transfected with or without Flag-OTUB1 and cultured under normoxia (21% O_2_) for 24 h, followed by treatment with DMSO or FG4592 (100 μM) for 8 h. Flag empty vector was used as a control. Two-way ANOVA analysis; Data show mean ± SD; Tukey’s multiple comparisons test; ns, not significant, *Adjusted *p* < 0.05, **Adjusted *p* < 0.01, ***Adjusted *p* < 0.001, ****Adjusted *p* < 0.0001; Data from 3 independent experiments. **E** Western blot analysis of indicated proteins in *VHL*-deficient or wildtype H1299 cells (*VHL*^−/−^ or *VHL*^+/+^) cultured under normoxia (21% O_2_). **F** qPCR analysis of *PDK1* and *PGK1* mRNA in *VHL*-deficient or wildtype H1299 cells (*VHL*^−/−^ or *VHL*^+/+^) cultured under normoxia (21% O_2_). Data show mean ± SD; Student’s two tailed *t*-test; ***p < 0.001; Data from 3 independent experiments. **G** qPCR analysis of *PDK1*, *PGK1*, *LDHA*, *VEGF* and *PKM2* mRNA in *VHL*-deficient H1299 cells (*VHL*^−/−^) transfected with or without Flag-OTUB1 and cultured under normoxia (21% O_2_) for 24 h. Flag empty vector was used as a control. Data show mean ± SD; Student’s two tailed *t*-test; ***p < 0.001; Data from 3 independent experiments. **H** Western blot analysis of indicated proteins in *FIH*-deficient or wildtype HEK293T cells (*FIH*^−/−^ or *FIH*^+/+^) cultured under normoxia (21% O_2_). **I**–**K** qPCR analysis of *PDK1* (**I**), *PGK1* (**J**) and *GLUT1* (**K**) mRNA in *FIH*-deficient HEK293T cells (*FIH* ^−/−^) transfected with or without Flag-OTUB1 and cultured under normoxia (21% O_2_) or hypoxia (1% O_2_) for 24 h. Flag empty vector was used as a control. Two-way ANOVA analysis; Data show mean ± SD; Tukey’s multiple comparisons test; ns, not significant, ****Adjusted *p* < 0.0001; Data from 3 independent experiments.

Given that PHDs-mediated HIF-α hydroxylation represents a main regulatory manner of HIF function [1, 21–23], we next sought to determine whether the upregulation of HIF activation by OTUB1 was dependent on PHDs. When H1299 cells were treated with the proline hydroxylase inhibitors, DMOG or FG4592 [70], overexpression of *OTUB1* still promoted expression of *PDK1*, *PGK1* or *BNIP3* (Fig. 3A–D). In contrast, expression of *PDK1, GLUT1*, or *BNIP3* was lower in *OTUB1*^−/−^ H1299 cells compared to *OTUB1*^+/+^ H1299 cells when either DMOG or FG4592 was added to cells (Fig. S5A–D). Furthermore, we examined whether the upregulation of HIF activation by OTUB1 was dependent on VHL. Knockout of *VHL* in H1299 cells caused an increased protein level of HIF-1α and an increased mRNA level of *PDK1* and *PGK1* under normoxia (Fig. 3E, F), indicating that *VHL* was disrupted efficiently in H1299 cells. Overexpression of *OTUB1* enhanced expression of *PDK1, PGK1, LDHA, VEGF*, and *PKM2* in *VHL*^−/−^ H1299 cells (Fig. 3G). Considering that FIH modulates OTUB1 activity, we subsequently examined whether the upregulation of HIF activation by OTUB1 is dependent on FIH. In *FIH*-deficient HEK293T cells (Fig. 3H), overexpression of *OTUB1* still enhanced expression of *PDK1, PGK1* and *GLUT1* under hypoxia (1% O_2_) (Fig. 3I–K). These data suggest that the enhancement of OTUB1 on HIF activity is independent of both PHDs/VHL and FIH.

### OTUB1 interacts with HIF-1α

**Fig. 4:**
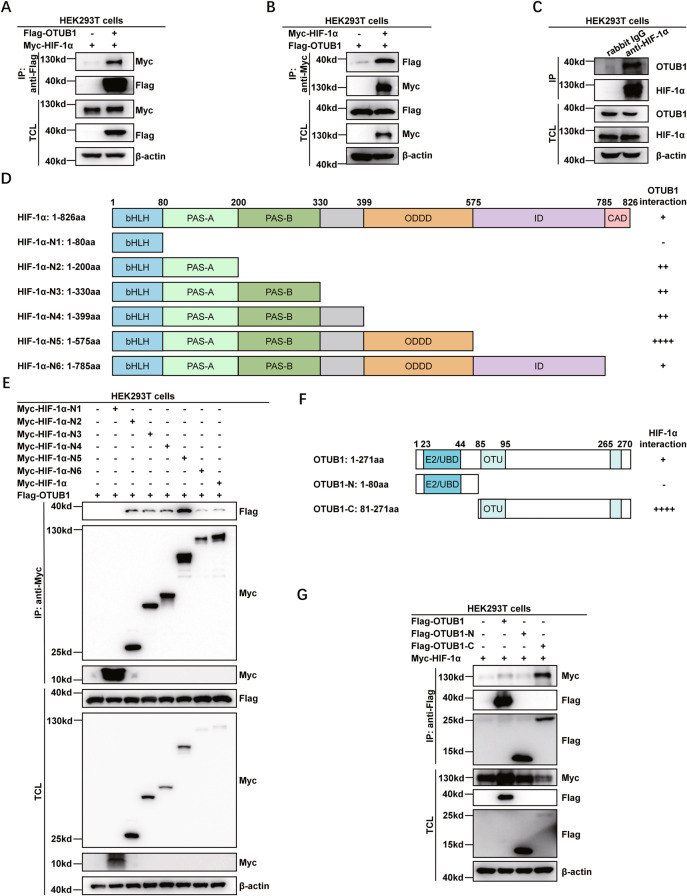
OTUB1 interacts with HIF-1α. **A**, **B** Co-immunoprecipitation of Flag-OTUB1 with Myc-HIF-1α and vice versa. HEK293T cells were co-transfected with indicated plasmids and cultured under normoxia (21% O_2_) for 24 h. Anti-Flag (**A**) or anti-Myc antibody-conjugated agarose beads (**B**) were used for immunoprecipitation, and the interaction was detected by immunoblotting with the indicated antibodies. **C** Endogenous interaction between OTUB1 and HIF-1α. HEK293T cells were cultured under hypoxia (1% O_2_) for 4 h. Anti-HIF-1α antibody was used for immunoprecipitation, and normal rabbit IgG was used as a control. **D** Schematic of HIF-1α domains interacted with OTUB1. The interaction is indicated by plus (+) sign. **E** Co-immunoprecipitation analysis of Flag-OTUB1 with Myc-HIF-1α-truncated mutants. HEK293T cells were co-transfected with the indicated plasmids and cultured under normoxia (21% O_2_) for 24 h. Anti-Myc antibody-conjugated agarose beads were used for immunoprecipitation, and the interaction was analyzed by immunoblotting with the indicated antibodies. Myc-HIF-1α fragments (HIF-1α-N1, 1–80 aa; HIF-1α-N2, 1–200 aa; HIF-1α-N3, 1–330 aa; HIF-1α-N4, 1–399 aa; HIF-1α-N5, 1–575 aa; HIF-1α-N6, 1–785 aa). **F** Schematic of OTUB1 domains interacted with HIF-1α. The interaction is indicated by plus (+) sign. **G** Co-immunoprecipitation analysis of Myc-HIF-1α with Flag-OTUB1-truncated mutants. HEK293T cells were co-transfected with the indicated plasmids and cultured under normoxia (21% O_2_) for 24 h. Anti-Flag antibody-conjugated agarose beads were used for immunoprecipitation, and the interaction was analyzed by immunoblotting with the indicated antibodies. Flag-OTUB1 fragments (OTUB1-N, 1–80 aa; OTUB1-C, 81–271 aa).

To determine the effect of OTUB1 on HIF activity mechanistically, we next examined whether OTUB1 interacts with HIF-1α. Ectopic expression of OTUB1 interacted with ectopically expressed HIF-1α in HEK293T cells and vice versa (Fig. 4A, B). In HEK293T cells, endogenous OTUB1 associated with endogenous HIF-1α (Fig. 4C). Domain mapping indicated that ODDD domain, ID and CAD domains of HIF-1α had highest binding capability for OTUB1, but the N-terminal domain (bHLH) did not bind to OTUB1 and was not required for the interaction between OTUB1 and HIF-1α (Fig. 4D, E, S6C, D). In *HIF-1β*-deficient H1299 cells (*HIF-1β*^−/−^), ectopic expression of OTUB1 interacted with ectopically expressed HIF-1α, indicating that hetero-dimerization of HIF-1α and HIF-1β is not required for the interaction between OTUB1 and HIF-1α (Fig. S6A, B). In addition, it appeared that the N-terminal domain (E2/UBD) of OTUB1 did not bind to HIF-1α, while the C-terminal domain of OTUB1 had highest binding capability for HIF-1α (Fig. 4F, G). These data suggest that OTUB1 might argument hypoxia signaling through enhancing HIF-1α function via protein interaction.

### OTUB1 stabilizes HIF-1α by inhibiting its ubiquitination

**Fig. 5:**
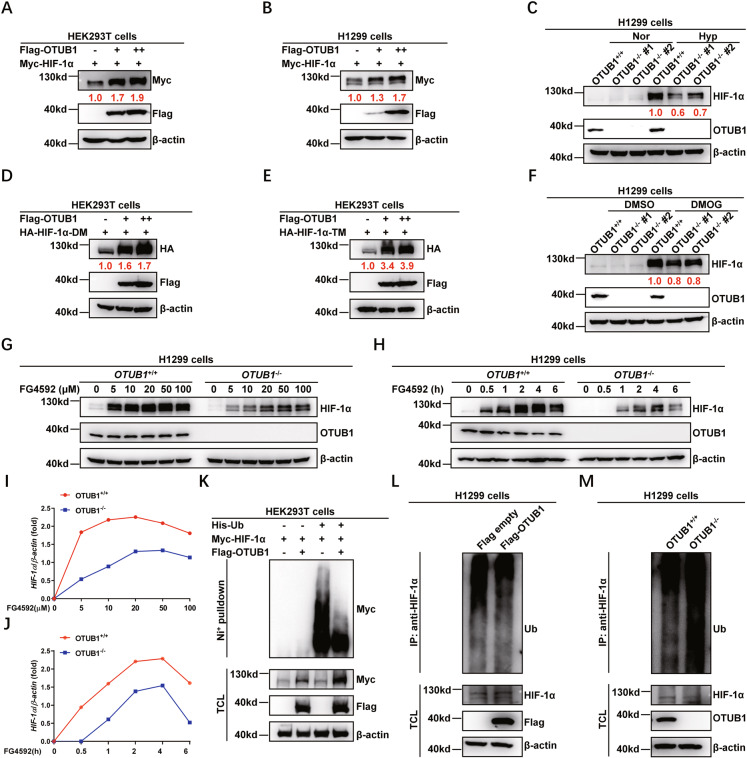
OTUB1 stabilizes HIF-1α by inhibiting ubiquitination of HIF-1α. **A** Immunoblotting of exogenous Myc-HIF-1α expression in HEK293T cells transfected with an increasing amount of Flag-OTUB1 expression plasmid. **B** Immunoblotting of exogenous Myc-HIF-1α expression in H1299 cells transfected with an increasing amount of Flag-OTUB1 expression plasmid. **C** Immunoblotting of endogenous HIF-1α expression in WT or two *OTUB1*-deficient H1299 cell lines (#1 and #2) cultured under normoxia (21% O_2_) or hypoxia (1% O_2_) for 4 h. **D** Immunoblotting of exogenous HA-HIF-1α-DM (encoding the double mutant of HIF-1α [P402A/P564A]) expression in HEK293T cells transfected with an increasing amount of Flag-OTUB1 expression plasmid. **E** Immunoblotting of exogenous HA-HIF-1α-TM (encoding the triple mutant of HIF-1α [P402A/P564A/N803A]) expression in HEK293T cells transfected with an increasing amount of Flag-OTUB1 expression plasmid. **F** Immunoblotting of endogenous HIF-1α expression in WT or two *OTUB1*-deficient H1299 cell lines treated with DMSO (as a control) or DMOG (1 mM) for 6 h. **G** Immunoblotting of endogenous HIF-1α expression in WT or *OTUB1*-deficient H1299 (*OTUB1*^+/+^ or *OTUB1*^−/−^) cells treated with an increasing amount of FG4592 for 6 h. **H** Immunoblotting of endogenous HIF-1α expression in WT or *OTUB1*-deficient H1299 (*OTUB1*^+/+^ or *OTUB1*^−/−^) cells treated with an increasing time of FG4592 (100 μM). **I** The relative intensities of HIF-1α in **G** determined by normalizing the intensities of HIF-1α to the intensities of β-actin. **J** The relative intensities of HIF-1α in **H** determined by normalizing the intensities of HIF-1α to the intensities of β-actin. **K** HIF-1α ubiquitination in HEK293T cells transfected with indicated plasmids for 24 h. **L** Endogenous HIF-1α ubiquitination in H1299 cells transfected with indicated plasmids and cultured under normoxia (21% O_2_) for 24 h, followed by MG-132 (20 μM) treatment for 6~8 h. **M** Endogenous HIF-1α ubiquitination in WT or OTUB1-deficient H1299 (*OTUB1*^+/+^ or *OTUB1*^−/−^) cells cultured under normoxia (21% O_2_) and treated with MG-132 (20 μM) for 6~8 h.

In analyzing the role of OTUB1 in the hypoxia signaling pathway, we examined the effect of OTUB1 on HIF-1α protein level and found that OTUB1 could upregulate the protein level of HIF-1α (Fig. S4E-G, I). Given that OTUB1 serves as a deubiquitinase, we sought to determine whether OTUB1 can directly affect HIF-1α protein stability. Ectopic expression of *OTUB1* in HEK293T cells increased protein level of co-transfected HIF-1α in a dose-dependent manner (Fig. 5A). Similar result was obtained in H1299 cells (Fig. 5B). By contrast, disruption of *OTUB1* in H1299 cells caused a reduction of endogenous HIF-1α protein under hypoxia (1% O_2_) (Fig. 5C). However, OTUB1 had no significant effect on the mRNA level of HIF-1α (Fig. S7A, C).

Furthermore, ectopic expression of *OTUB1* also increased protein levels of co-transfected HIF-1α-DM (in which two Proline residues [P402 and P564] are mutated to Alanine residues) and HIF-1α-TM (in which two Proline residues [P402 and P564] and one Asparagine residue [N803] is mutated to Alanine residue) (Fig. 5D, E), suggesting that the stabilization of HIF-1α protein by OTUB1 might be independent of hydroxylation mediated by PHDs and FIH. In agreement, when DMOG was added, endogenous HIF-1α protein level in *OTUB1*-knocked out H1299 cells (*OTUB1*^−/−^ #1 and *OTUB1*^−/−^ #2) was lower than that in wildtype H1299 cells (*OTUB1*^+/+^) (Fig. 5F). Of note, after treated with FG4592 at an increased concentrations or extended time periods, endogenous HIF-1α protein level in *OTUB1*^−/−^ H1299 cells kept lower than that in *OTUB1*^+/+^ H1299 cells (Fig. 5G–J). However, unlike the effect on HIF-1α protein level, OTUB1 did not have a significant effect either on protein levels of VHL and FIH (Fig. S7E, F), or on the mRNA of VHL (Fig. S7B, D).These data indicate that OTUB1 stabilizes HIF-1α independent of HIF-1α hydroxylation mediated by both PHDs and FIH, consistent with the results obtained in the above assays for the effect of OTUB1 on HIF activity.

Besides, MG-132 treatment could block OTUB1’s effect on protein levels of HIF-1α (Fig. S7G). So we examined whether OTUB1 could inhibit ubiquitination of HIF-1α subsequently. Overexpression of *OTUB1* in HEK293T cells suppressed ubiquitination of co-transfected HIF-1α (Fig. 5K). Moreover, ectopic expression of *OTUB1* in H1299 cells suppressed ubiquitination of endogenous HIF-1α (Fig. 5L). By contrast, knockout of *OTUB1* in H1299 (*OTUB1*^−/−^) enhanced ubiquitination of HIF-1α (Fig. 5M).

Taken together, these data suggest that OTUB1 stabilizes HIF-1α by inhibiting ubiquitination of HIF-1α.

### OTUB1 inhibits K48-linked ubiquitination of HIF-1α via its non-canonical inhibition of ubiquitination activity

**Fig. 6:**
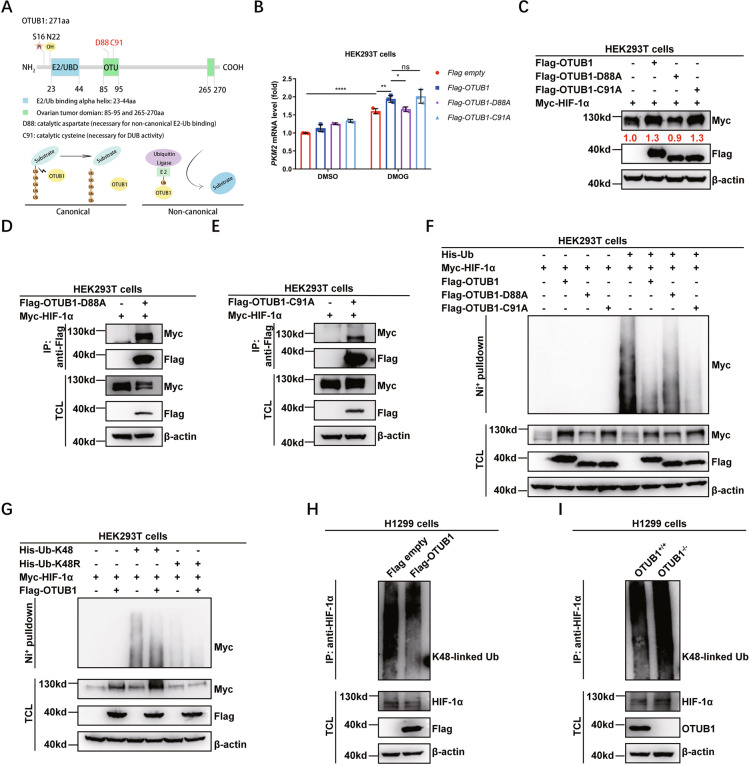
OTUB1 inhibits K48-linked polyubiquitination of HIF-1α dependent of its non-canonical activity. **A** Schematic of the domain architecture of OTUB1, showing its E2-binding/ubiquitin binding domain (UBD) region (E2-UBD), ovarian tumor domain (OTU), catalytic sites and modification sites. The working model shows the canonical enzymatic activity and non-canonical activity mediated by OTUB1. **B** qPCR analysis of *PKM2* mRNA in HEK293T cells transfected with Flag-OTUB1 or its mutants with the treatment of DMSO (as a control) or DMOG (1 mM) for 6 h. Flag empty vector was used as a control. Two-way ANOVA analysis; Data show mean ± SD; Tukey’s multiple comparisons test; ns, not significant, ****Adjusted *p* < 0.0001; Data from 3 independent experiments. **C** Immunoblotting of exogenous Myc-HIF-1α expression in HEK293T cells transfected with expression plasmid encoding Flag-OTUB1 or its enzymatically deficient mutants. **D** Co-immunoprecipitation of Flag-OTUB1-D88A with Myc-HIF-1α. HEK293T cells were co-transfected with indicated plasmids for 24 h. Anti-Flag antibody-conjugated agarose beads were used for immunoprecipitation, and the interaction was detected by immunoblotting with the indicated antibodies. **E** Co-immunoprecipitation of Flag-OTUB1-C91A with Myc-HIF-1α. HEK293T cells were co-transfected with indicated plasmids for 24 h. Anti-Flag antibody-conjugated agarose beads were used for immunoprecipitation, and the interaction was detected by immunoblotting with the indicated antibodies. **F** HIF-1α ubiquitination in HEK293T cells transfected with Myc-HIF-1α, His-Ub (His empty vector was used as a control), together with Flag-OTUB1, Flag-OTUB1-D88A or Flag-OTUB1-C91A (Flag empty vector was used as a control) for 24 h. **G** HIF-1α ubiquitination in HEK293T cells transfected with indicated plasmids for 24 h. **H** Endogenous HIF-1α ubiquitination in H1299 cells transfected with indicated plasmids for 24 h, followed by MG-132 (20 μM) treatment for 6~8 h. **I** Endogenous HIF-1α ubiquitination in WT or *OTUB1*-deficient H1299 (*OTUB1*^+/+^ or *OTUB1*^−/−^) cells treated with MG-132 (20 μM) for 6~8 h.

OTUB1 was previously identified as a member of the ovarian tumor domain containing a superfamily of proteases that has two distinct activities: canonical enzymatic activity for K48-linked polyubiquitin hydrolysis and non-canonical activity for the formation of E2-repressive complex (Fig. 6A) [49, 50, 71]. To determine which activity is required for OTUB1’s effect on HIF-1α, we constructed two mutants: C91A (defective in canonical deubiquitinase activity) and D88A (defective in binding to E2 enzymes), and examined their effects. In the presence of DMOG, overexpression of OTUB1-D88A in HEK293T cells had no effect on the induction of *PKM2* mRNA level, but overexpression of OTUB1-C91A still increased *PKM2* mRNA level, similar to that of wildtype OTUB1 (Fig. 6B). Moreover, overexpression of OTUB1-D88A in HEK293T cells did not stabilize co-transfected HIF-1α protein level, but overexpression of OTUB1-C91A still stabilized co-transfected HIF-1α protein level, similar to that of wildtype OTUB (Fig. 6C). However, two mutants could interact with HIF-1α as well (Fig. 6D, E).

Subsequently, we examined the effect of two mutants on ubiquitination of HIF-1α. As shown in Fig. 6F, the ubiquitination inhibition of OTUB1-D88A on HIF-1α is not as dramatic as that of wildtype OTUB1 and OTUB1-C91A (Fig. 6F). As expected, overexpression of *OTUB1* reduced K48 linkage-specific ubiquitination of HIF-1α (Fig. 6G). Furthermore, we confirmed that overexpression of *OTUB1* in H1299 cells inhibited endogenous K48-linked ubiquitination of HIF-1α (Fig. 6H). By contrast, knockout of *OTUB1* in H1299 cells enhanced K48-linked ubiquitination of HIF-1α (Fig. 6I).

Together, these data suggest that OTUB1 inhibits K48-linked ubiquitination of HIF-1α via its non-canonical inhibition of ubiquitination activity.

### OTUB1 facilitates hypoxia adaptation

**Fig. 7:**
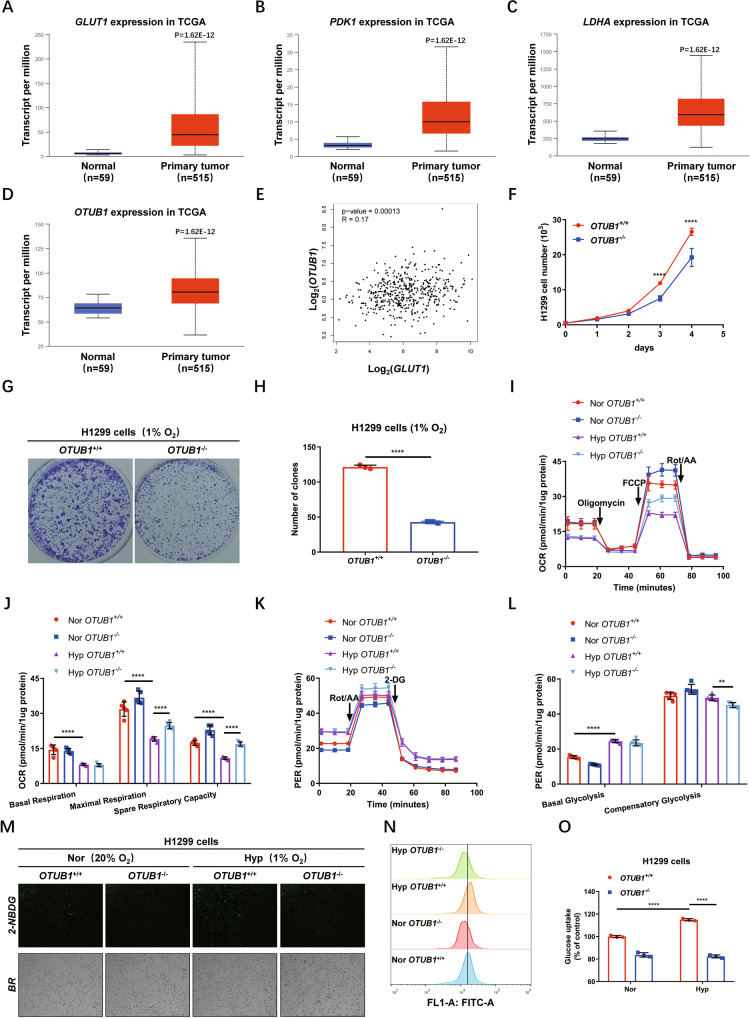
OTUB1 facilitates hypoxia adaptation. **A**–**D** Comparison of *GLUT1* (**A**), *PDK1* (**B**), *LDHA* (**C**) and *OTUB1* (**D**) expressions in lung cancer tissues (*n* = 515) and in the adjacent normal tissues (*n* = 59). *GLUT1* (**A**), *PDK1* (**B**)*, LDHA* (**C**) and *OTUB1* (**D**) mRNA levels in tumors were higher than in normal tissues, as determined by the student’s *t* test. The data were obtained from the Cancer Genome Atlas (TCGA) data (https://cancergenome.nih.gov/) and analyzed by the online tool UALCAN (http://ualcan.path.uab.edu/). **E** Linear regression of *OTUB1* and *GLUT1* across the panels of normal and lung cancer samples described in **A**, **D**, was generated by the online analysis tool GEPIA (http://gepia.cancer-pku.cn/) based on TCGA data (https://cancergenome.nih.gov/). **F** Growth curves of WT or *OTUB1*-deficient H1299 (*OTUB1*^+/+^ or *OTUB1*^−/−^) cells (*n* = 5) cultured for the indicated days by CCK-8 assay. **G**, **H** Colony formation of WT or *OTUB1*-deficient H1299 (*OTUB1*^+/+^ or *OTUB1*^−/−^) cells (*n* = 3) cultured under hypoxia for the indicated days. **I**, **J** Oxygen consumption rate (OCR) changes in wildtype (WT) or *OTUB1*-deficient H1299 (*OTUB1*^+/+^ or *OTUB1*^−/−^) cells (*n* = 5) under normoxia (Nor) or hypoxia (Hyp) measured by Seahorse XFe24 Extracellular Flux Analyzer (**I**). Statistics of basal respiration, maximal respiration, and spare respiratory capacity were presented in **J**. **K**, **L** Proton efflux rate (PER) changes in WT or *OTUB1*-deficient H1299 (*OTUB1*^+/+^ or *OTUB1*^−/−^) cells (*n* = 5) under normoxia (Nor) or hypoxia (Hyp) measured by Seahorse XFe24 Extracellular Flux Analyzer. Statistics of basal glycolysis and compensatory glycolysis were presented in **L**. **M**–**O** Glucose uptake in WT or *OTUB1*-deficient H1299 (*OTUB1*^+/+^ or *OTUB1*^−/−^) cells (*n* = 3) under normoxia or hypoxia analyzed using fluorescent glucose analog 2-NBDG and detected by fluorescence microscopy (M), and flow cytometry analysis (**N**, **O**).

Given the importance of hypoxia signaling in cancer initiation and progression [1, 22, 23], to get insights into the biological function of *OTUB1* mediated by its effect on hypoxia signaling, we sought to look into its expression in cancer tissues. Based on the Cancer Genome Atlas (TCGA) database (https://cancergenome.nih.gov/), we noticed that expression of *OTUB1* in lung cancer tissues was higher than in normal tissues, similar to *GLUT1, PDK1* and *LDHA* (Fig. 7A–D). In addition, the correlation of expression of *OTUB1* and *GLUT1, LDHA or PGK1* was validated (Figs. 7E, S8A, B). Consistently, *OTUB1*-deficient H1299 cells (*OTUB1*^−/−^) proliferated much slower than *OTUB1*-intact H1299 cells (*OTUB1*^+/+^) (Fig. 7F–H).

It has been well-established that HIF-1 is a master regulator for metabolic adaptation under hypoxia [72–74]. Subsequently, we compared the oxygen consumption rate between wildtype (*OTUB1*^+/+^) and *OTUB1*-deficient H1299 (*OTUB1*^−/−^) cells under normoxia or hypoxia based on mitochondrial stress test. Under hypoxia, the maximal respiration and spare respiratory capacity of *OTUB1*^−/−^ H1299 cells was higher than that of *OTUB1*^+/+^H1299 cells (Fig. 7I–J). We next determined the effect of *OTUB1* on glycolysis via the proton efflux rate measurement. Loss of *OTUB1* in H1299 cells led to decreased compensatory glycolysis significantly compared to wildtype H1299 cells under hypoxia (Fig. 7K, L). Moreover, Loss of *OTUB1* in H1299 cells diminished glucose uptake (Fig. 7M–O).

Collectively, these data suggest that OTUB1 facilitates hypoxia adaptation, which might benefit cancer progression.

In order to determine whether the formation of FIH and OTUB1 heterodimers could affect OTUB1’s regulation on hypoxia signaling, we used FG4592 to stabilize HIF-1α under normoxia. Upon FG4592 treatment, FIH had no obvious effect on HIF-1α’s protein level, while OTUB1 could stabilize HIF-1α. However, FIH and OTUB1 co-expression could eliminate the stabilization of HIF-1α by OTUB1 (Fig. S9A, B).

**Fig. 8:**
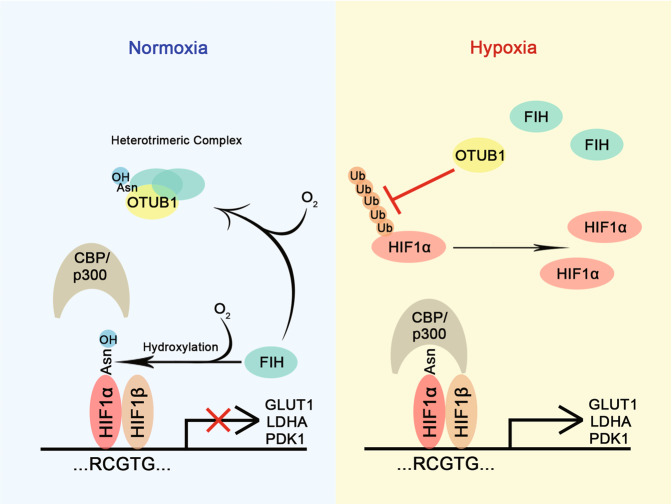
Schematic of OTUB1 action in the regulation of hypoxia signaling. Under normoxia, OTUB1 forms a complex with FIH dependent of oxygen and loses its non-canonical inhibition of ubiquitination activity. Under hypoxia, OTUB1 is dissociated from FIH, which inhibits K48-linked polyubiquitination of HIF-1α. As a result, HIF-1α is stabilized and transactivates downstream genes.

Based on the above observations, a working model for *OTUB1* in the regulation of hypoxia signaling is proposed (Fig. 8).

## Discussion

The regulation of HIF-1α protein stability under normoxia and hypoxia is well-defined [1]. Under normoxia, PHDs/VHL system keeps HIF-1α protein at almost undetectable level, but, under hypoxia, because PHDs lose enzymatic activity, HIF-1α protein is stabilized and acts its function [1, 75, 76]. Even though the regulation of HIF-1α protein stability by PHDs/VHL accounts for a major regulatory manner for hypoxia signaling, the factors other than PHDs/VHL might also be involved in the regulation of HIF-1α protein stability, particularly some deubiquitinases [25]. Therefore, some efforts have been taken to identify deubiquitinases for regulating HIF [25, 31, 32, 35, 36]. Actually, whether HIF-1α protein stability could be regulated under hypoxia is still a puzzle. In this study, we identified that OTUB1 is involved in regulating HIF-1α activity independent of PHDs/VHL and FIH, and disruption of OTUB1 reduces HIF-1α protein under hypoxia. However, the effect of OTUB1 on HIF-1α function under normoxia conditions was not as consistent as that under hypoxia-mimicking conditions and for that reason the effect of OTUB1 on HIF-1α function in normoxic conditions was not studied. Given that the non-canonical activity of OTUB1 is suppressed by FIH under normoxia [59] and OTUB1 regulates HIF-1α via its non-canonical inhibition of ubiquitination, OTUB1 might contribute to the stabilization of HIF-1α protein under hypoxia. In fact, in *VHL*-deficient tumors, HIF-1α protein level keeps at very high level, which is considered to be a major cause of cancer progression [75, 76] [36]. Here, we find that OTUB1 is involved in HIF-1α stabilization independent of *VHL*, so, OTUB1 might be a therapeutic target for the treatment of VHL-deficient tumors.

Even though several deubiquitinases of HIF-α have been identified so far, actually, how they act roles for regulating HIF activity under hypoxia is still not well-understood [25, 26, 31, 35, 36]. Here, inferred from the modulation of OTUB1 by FIH, an important regulator of HIF pathway, we further identified when FIH loses its suppressive role on the non-canonical inhibition ubiquitination activity of OTUB1 under hypoxia, OTUB1 enhances HIF-1α activity, uncovering a connection among FIH/OTUB1/HIF-1α. This finding provides evidence for supporting multiple regulatory manners in hypoxia signaling pathway.

In this study, for the first time, we found that OTUB1, is involved in the regulation of hypoxia signaling. Given that importance of hypoxia signaling in metabolism, cancer progression, and other multiple physiological processes, to investigate the role and the underlying mechanism of OTUB1 in these processes will open a new window for understanding the physiological relevance of *OTUB1*.

## Supplementary information

Supplemental Figure Legends and Table

Supplemental Figure 1

Supplemental Figure 2

Supplemental Figure 3

Supplemental Figure 4

Supplemental Figure 5

Supplemental Figure 6

Supplemental Figure 7

Supplemental Figure 8

Supplemental Figure 9

Original Western Blots

Reproducibility checklist

## Data Availability

Further information and requests for resources and reagents should be directed to and will be fulfilled by Wuhan Xiao.
