# Supplementary material for: OTUB1 augments hypoxia signaling via its non-canonical ubiquitination inhibition of HIF-1α during hypoxia adaptation
Source: Cell Death Dis. 2022 Jun 22;13(6):560. doi: 10.1038/s41419-022-05008-z (PMC9217984; doi:10.1038/s41419-022-05008-z)
Supplement: Supplementary file 1 — Supplemental Figure Legends and Table [file 41419_2022_5008_MOESM1_ESM.docx]

**Supplementary information**

**Supplementary Data**

**Figure S1. Characterization of the FIH-OTUB1 conjugation.**

(A) Immunoblotting of the exogenous Flag-OTUB1, Myc-FIH or the heterodimer (composed of Flag-OTUB1 and Myc-FIH; indicated as “HD”) expression level in HEK293T cells co-transfected with the indicated plasmids.

(B) Immunoblotting of the exogenous Flag-OTUB1, Myc-FIH or the heterodimer (composed of Flag-OTUB1 and Myc-FIH; indicated as “HD”) expression level in HEK293T cells co-transfected with the indicated plasmids and cultured under hypoxia (1% O_2_) for an increasing time.

(C) Immunoblotting of the exogenous Flag-OTUB1 or Flag-OTUB1-N22A (encoding the hydroxylation site mutant of OTUB1 [N22A]), Myc-FIH or Myc-FIH-H199A (encoding the enzymatically deficient mutant of FIH [H199A]) or the heterodimer (composed of Flag-OTUB1 and Myc-FIH; indicated as “HD”) expression level in HEK293T cells co-transfected with the indicated plasmids and cultured under hypoxia (1% O_2_) for an increasing time.

**Figure S2. Hypoxia induces the expression of OTUB1.**

(A) qPCR analysis of *OTUB1*, *PDK1*, *PGK1*, *VEGF* and *LDHA* mRNA in H1299 cells cultured under normoxia (21% O_2_) or hypoxia (1% O_2_) for 24 h. Data show mean + SD; Student’s two tailed t-test; * p<0.05, ** p <0.01, *** p <0.001, **** p <0.0001; Data from 3 independent experiments.

(B) Immunoblotting of endogenous OTUB1 and HIF-1α expression in H1299 cells cultured under hypoxia (1% O_2_) for an increasing time.

**Figure S3. OTUB1 augments hypoxia signaling.**

(A-D) Quantitative real-time PCR (qPCR) analysis of *PDK1* (A), *LDHA* (B), *VEGF* (C), and *BNIP3* (D) mRNA in HEK293T cells transfected with or without Flag-OTUB1 and cultured under normoxia (21% O_2_) or hypoxia (1% O_2_) for 24 h. Flag empty vector was used as a control. Two-way ANOVA analysis; Data show mean + SD; Tukey's multiple comparisons test; ns, not significant, * Adjusted p<0.05, ** Adjusted p <0.01, *** Adjusted p <0.001, **** Adjusted p <0.0001; Data from 3 independent experiments.

(E) Immunoblotting of transfected Flag-OTUB1 expression in HEK293T cells.

(F) Immunoblotting of transfected Flag-OTUB1 expression in H1299 cells.

**Figure S4. OTUB1 deficiency downregulates the protein level of HIF-1α and reconstitution of OTUB1 activates hypoxia signaling.**

(A-D) qPCR analysis of *PDK1* (A), *PGK1* (B), *GLUT1* (C) and *LDHA* (D) mRNA in *OTUB1*-deficient H1299 cells (*OTUB1*^-/-^) transfected with or without Flag-OTUB1 and cultured under normoxia (21% O_2_) or hypoxia (1% O_2_) for 24 h. Flag empty vector was used as a control. Two-way ANOVA analysis; Data show mean + SD; Tukey's multiple comparisons test; ns, not significant, * Adjusted p<0.05, ** Adjusted p <0.01, *** Adjusted p <0.001, **** Adjusted p <0.0001; Data from 3 independent experiments.

(E) Immunoblotting of endogenous HIF-1α expression in WT or *OTUB1*-deficient H1299 (*OTUB1*^+/+^ or *OTUB1*^-/-^) cells under normoxia (21% O_2_) or hypoxia (1% O_2_) as indicated in Figure 2B to 2D.

(F) Immunoblotting of endogenous HIF-1α expression in WT or *OTUB1*-deficient H1299 (*OTUB1*^+/+^ or *OTUB1*^-/-^) cells treated with or without CoCl_2_ as indicated in Figure 2E to 2H.

(G) Immunoblotting of endogenous HIF-1α expression in WT or *OTUB1*-deficient H1299 (*OTUB1*^+/+^ or *OTUB1*^-/-^) cells treated with DMSO or DFX as indicated in Figure 2I to 2L.

(H) Immunoblotting of transfected Flag-OTUB1 expression in in *OTUB1*-deficient H1299 cells (*OTUB1*^-/-^).

(I) Immunoblotting of endogenous HIF-1α expression in *OTUB1*-deficient H1299 cells (*OTUB1*^-/-^) transfected with or without Flag-OTUB1 as indicated in Figure 2B to 2D.

**Figure S5. OTUB1 augments hypoxia signaling pathway independent of HIF-1α hydroxylation.**

(A, B) qPCR analysis of *PDK1* (A) and *GLUT1* (B) mRNA in *OTUB1*-deficient or wildtype H1299 cells (*OTUB1*^-/-^ or *OTUB1*^+/+^) treated with DMSO or DMOG (1mM) for 8 h.

(C, D) qPCR analysis of *GLUT1* (C) and *BNIP3* (D) mRNA in *OTUB1*-deficient or wildtype H1299 cells (*OTUB1*^-/-^ or *OTUB1*^+/+^) treated with DMSO or FG4592 (100μM) for 8 h.

Two-way ANOVA analysis; Data show mean + SD; Tukey's multiple comparisons test; Data from 3 independent experiments. ns, not significant, * Adjusted p<0.05, ** Adjusted p <0.01, *** Adjusted p <0.001, **** Adjusted p <0.0001.

**Figure S6. OTUB1 interacts with HIF-1α.**

(A) Immunoblotting of endogenous HIF-1β expression in *HIF-1β*-deficient or wildtype H1299 cells (*HIF-1β*^-/-^ or *HIF-1β*^+/+^).

(B) Co-immunoprecipitation of Flag-OTUB1 with Myc-HIF-1α in *HIF-1β*-deficient H1299 cells (*HIF-1β*^-/-^). The cells were co-transfected with indicated plasmids for 24 h. Anti-Flag antibody-conjugated agarose beads were used for immunoprecipitation, and the interaction was detected by immunoblotting with the indicated antibodies.

(C) Co-immunoprecipitation analysis of Flag-OTUB1 with Myc-HIF-1α-truncated mutants. HEK293T cells were co-transfected with the indicated plasmids. Anti-Myc antibody-conjugated agarose beads were used for immunoprecipitation, and the interaction was analyzed by immunoblotting with the indicated antibodies. Myc-HIF-1α fragments (HIF-1α-C1, 81–826 aa; HIF-1α-C2, 201–826 aa; HIF-1α-C3, 331–826 aa; HIF-1α-C4, 400–826 aa; HIF-1α-C5, 576–826 aa).

(D) Schematic of HIF-1α domains interacted with OTUB1. The interaction is indicated by plus (+) sign.

**Figure S7. OTUB1 upregulates HIF-1α expression at protein level.**

(A, B) qPCR analysis of *HIF-1α* (A), and *VHL* (B) mRNA in *OTUB1*-deficient or wildtype H1299 cells (*OTUB1*^-/-^ or *OTUB1*^+/+^) cultured under normoxia (21% O_2_) or hypoxia (1% O_2_) for 24 h.

(C, D) qPCR analysis of *HIF-1α* (C), and *VHL* (D) mRNA in *OTUB1*-deficient H1299 cells (*OTUB1*^-/-^) transfected with or without Flag-OTUB1 and cultured under normoxia (21% O_2_) or hypoxia (1% O_2_) for 24 h.

(E) Immunoblotting of endogenous HIF-1α, FIH, and VHL expression in *OTUB1*-deficient or wildtype H1299 cells (*OTUB1*^-/-^ or *OTUB1*^+/+^) treated with DMSO or DMOG (1 mM) for 8 h.

(F) Immunoblotting of endogenous HIF-1α, FIH, and VHL expression in *OTUB1*-deficient or wildtype H1299 cells (*OTUB1*^-/-^ or *OTUB1*^+/+^) treated with DMSO or FG4592 (100 μM) for 8 h.

(F) Immunoblotting of endogenous HIF-1α expression in *OTUB1*-deficient or wildtype H1299 cells (*OTUB1*^-/-^ or *OTUB1*^+/+^) cultured under hypoxia (1% O_2_) for 6 h, followed by treatment with DMSO or MG-132 (20 μM) for 6 h.

**Figure S8. OTUB1 expression correlates with HIF-1α target genes in lung cancer tissues.**

(A, B) Linear regression of *OTUB1* and *LDHA* or *PGK1* across the panels of normal and lung cancer samples described in Figure 7A to 7D was generated by the online analysis tool GEPIA (http://gepia.cancer-pku.cn/) based on TCGA data (https://cancergenome.nih.gov/).

**Figure S9. The formation of FIH and OTUB1 heterodimers could eliminate the stabilization of HIF-1α by OTUB1.**

(A) Immunoblotting of endogenous HIF-1α expression in HEK293T cells transfected with an increasing amount of Myc-FIH, followed by treatment with FG4592 (100 μM) for 6 h.

(B) Immunoblotting of endogenous HIF-1α expression in HEK293T cells transfected with Flag-OTUB1 together with Myc-FIH or Myc empty as a control, followed by treatment with FG4592 (100 μM) for 6 h.

**Supplementary Table S1. The primer sequences**

| Primers | Sequence (5’ to 3’) |
| --- | --- |
| Human-*18SrRNA*-RT-F | GAATTCCCAGTAAGTGGGGG |
| Human-*18SrRNA* -RT-R | GGGCAGGGACTTAATCAACG |
| Human-*PGK1*-RT-F | TGGCTTCTGGCATACCTGCT |
| Human-*PGK1*-RT-R | GCTGCTTTCAGGACCACAGCT |
| Human-*LDHA*-RT-F | AGGAAGAACAGACCCCCCAG |
| Human-*LDHA*-RT-R | CAGCACCAACCCCAACAACT |
| Human-*GLUT1*-RT-F | TGTGGGCCTTTTCGTTAACC |
| Human-*GLUT1*-RT-R | ATCATCAGCATTGAATTCCGC |
| Human-*VEGF*-RT-F | TGTGCCCACTGAGGAGTCC |
| Human-*VEGF*-RT-R | GGTTTGATCCGCATAATCTGC |
| Human-*PDK1*-RT-F | GATGCTAAAGCTATTTATGACT |
| Human-*PDK1*-RT-R | GGAATGACATCATTGTGTCGG |
| Human-*PKM2*-RT-F | GCCTGCTGTGTCGGAGAAG |
| Human-*PKM2*-RT-R | CAGATGCCTTGCGGATGAATG |
| Human-*BNIP3*-RT-F | CTTCCATCTCTGCTGCTCTC |
| Human-*BNIP3*-RT-R | GTAATCCACTAACGAACCAAGTC |
| Human-*HIF-1α*-RT-F | TTTTACCATGCCCCAGATTCA |
| Human-*HIF-1α*-RT-R | AGTGCTTCCATCGGAAGGACT |
| Human-*VHL*-RT-F | CGGACAGCCTATTTTTGCCAAT |
| Human-*VHL*-RT-R | ATGTTTGCCCCTAAACATCACA |
| Human-*OTUB1*-RT-F | TTTCTATCGGGCTTTCGGA |
| Human-*OTUB1*-RT-R | TCGGAGGTGCTCTGGTCAT |
