# Supplementary figures and images for: OTUB1 augments hypoxia signaling via its non-canonical ubiquitination inhibition of HIF-1α during hypoxia adaptation
Source: Cell Death Dis. 2022 Jun 22;13(6):560. doi: 10.1038/s41419-022-05008-z (PMC9217984; doi:10.1038/s41419-022-05008-z)

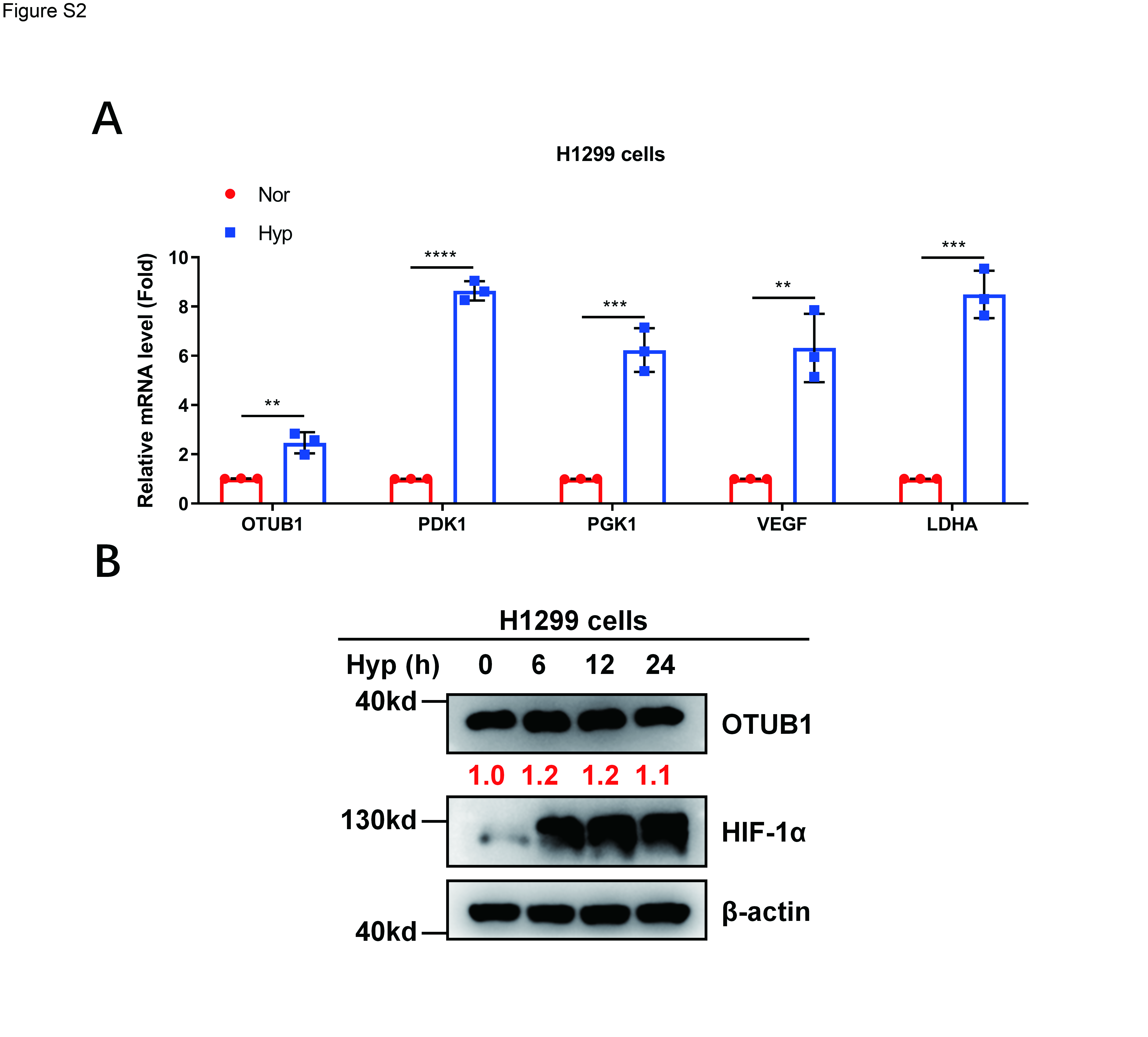

Supplement: Supplementary file 3 — Supplemental Figure 2 [file 41419_2022_5008_MOESM3_ESM.tif]

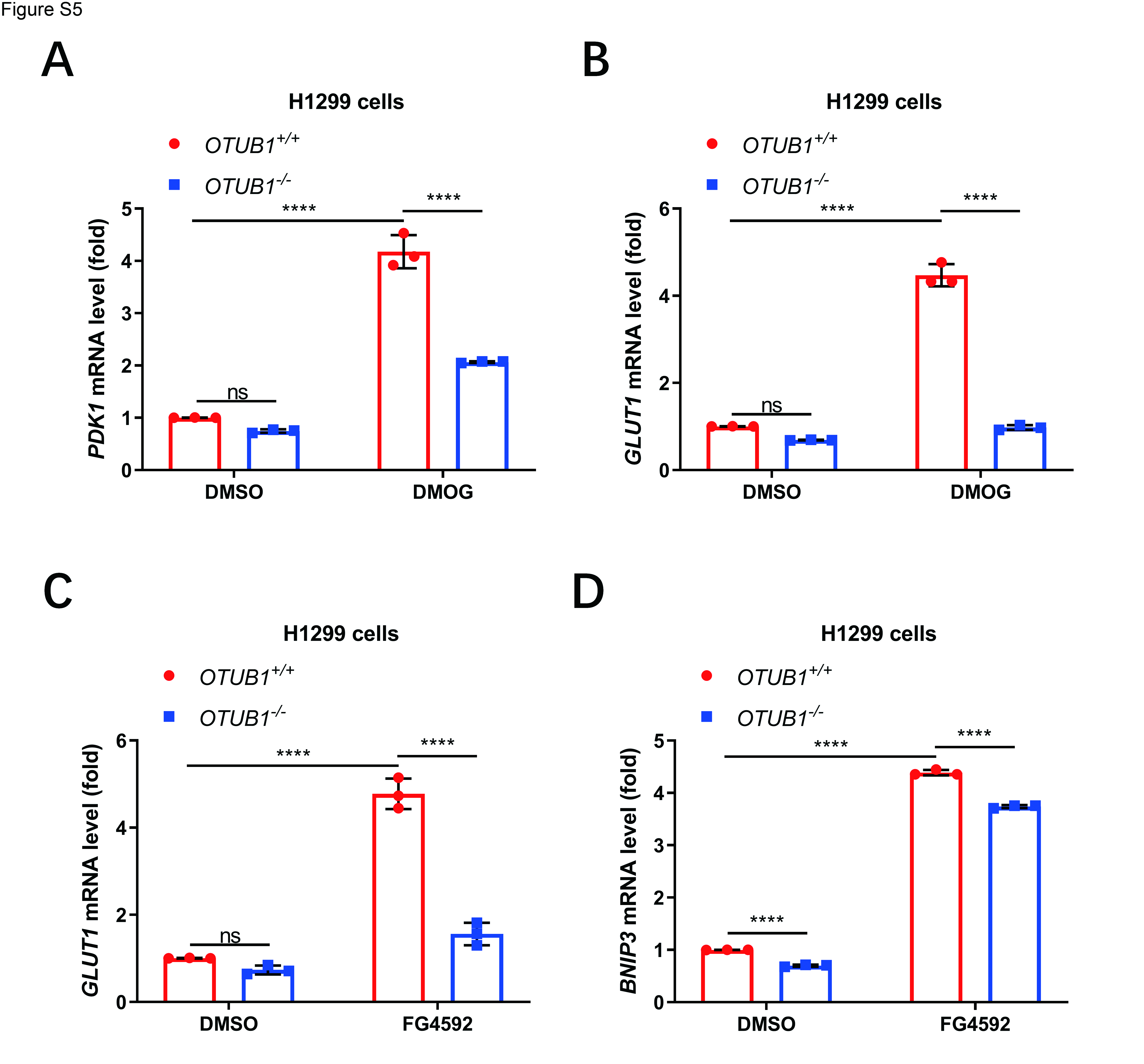

Supplement: Supplementary file 6 — Supplemental Figure 5 [file 41419_2022_5008_MOESM6_ESM.tif]

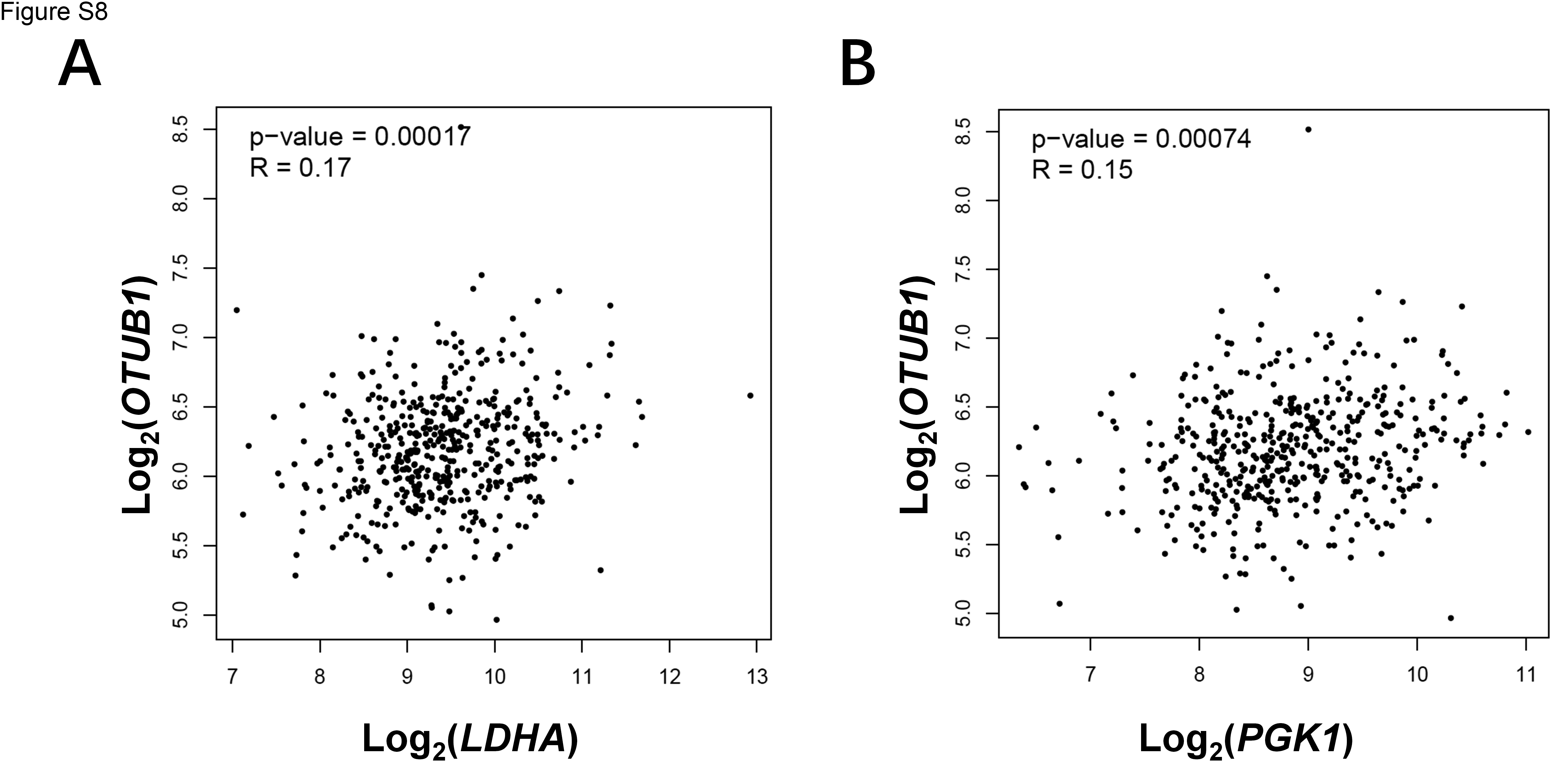

Supplement: Supplementary file 9 — Supplemental Figure 8 [file 41419_2022_5008_MOESM9_ESM.tif]

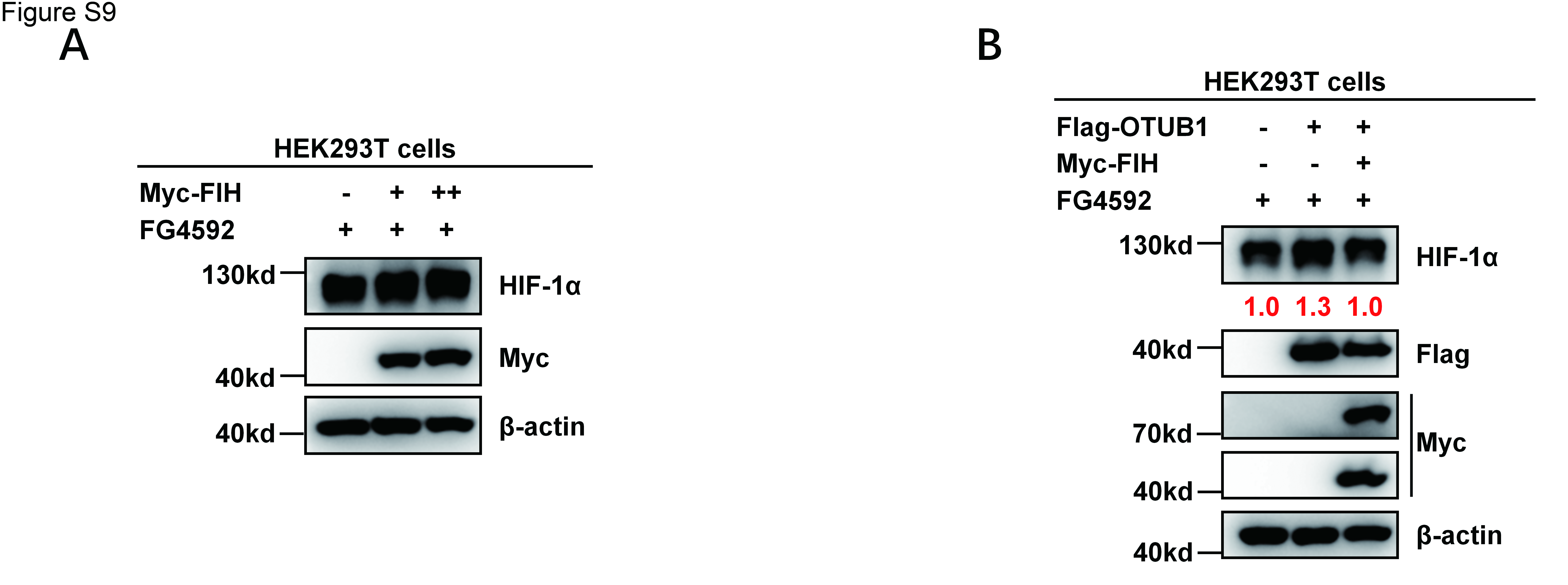

Supplement: Supplementary file 10 — Supplemental Figure 9 [file 41419_2022_5008_MOESM10_ESM.tif]
