# Supplementary material for: OTUB1 augments hypoxia signaling via its non-canonical ubiquitination inhibition of HIF-1α during hypoxia adaptation
Source: Cell Death Dis. 2022 Jun 22;13(6):560. doi: 10.1038/s41419-022-05008-z (PMC9217984; doi:10.1038/s41419-022-05008-z)

Original Western blot

Fig. 2A

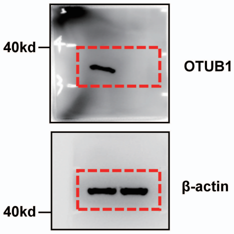

Fig. 3E

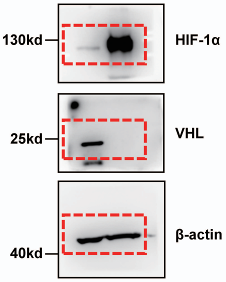

Fig. 3H

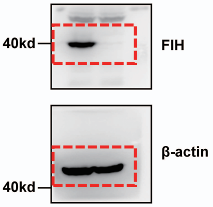

Fig. 4A

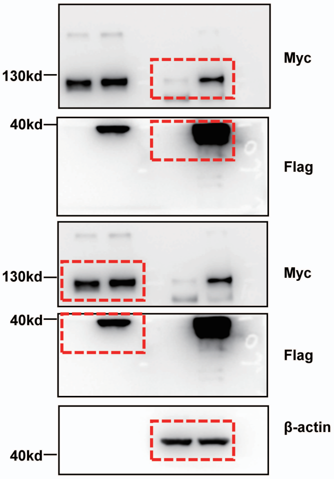

Fig. 4B

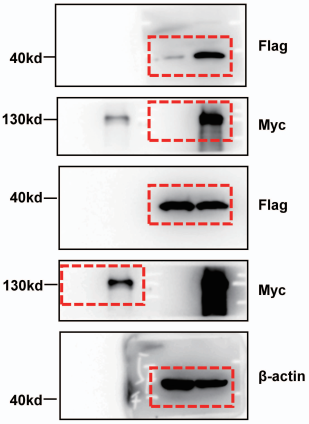

Fig. 4C

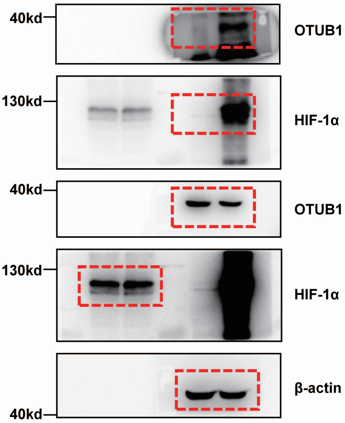

Fig. 4E

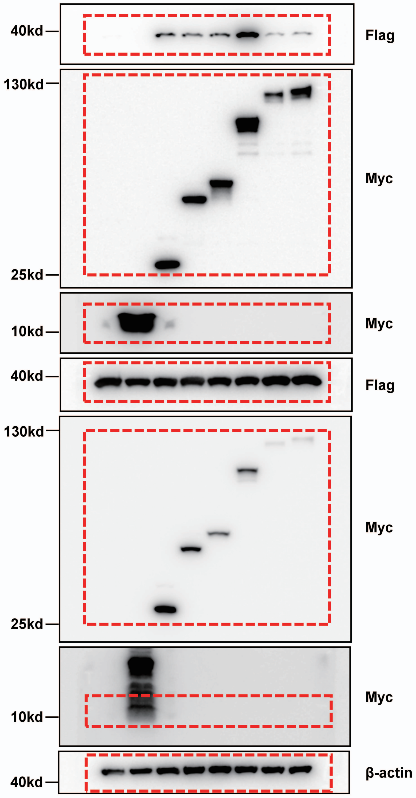

Fig. 4G

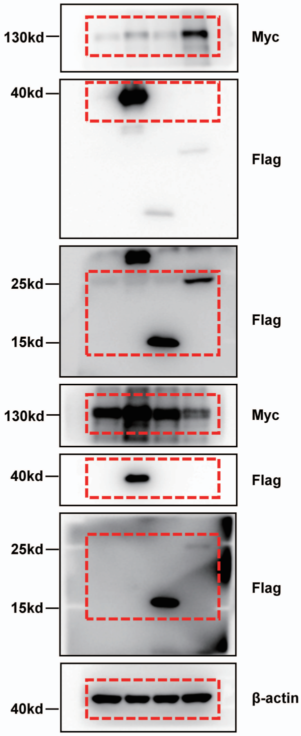

Original Western blot

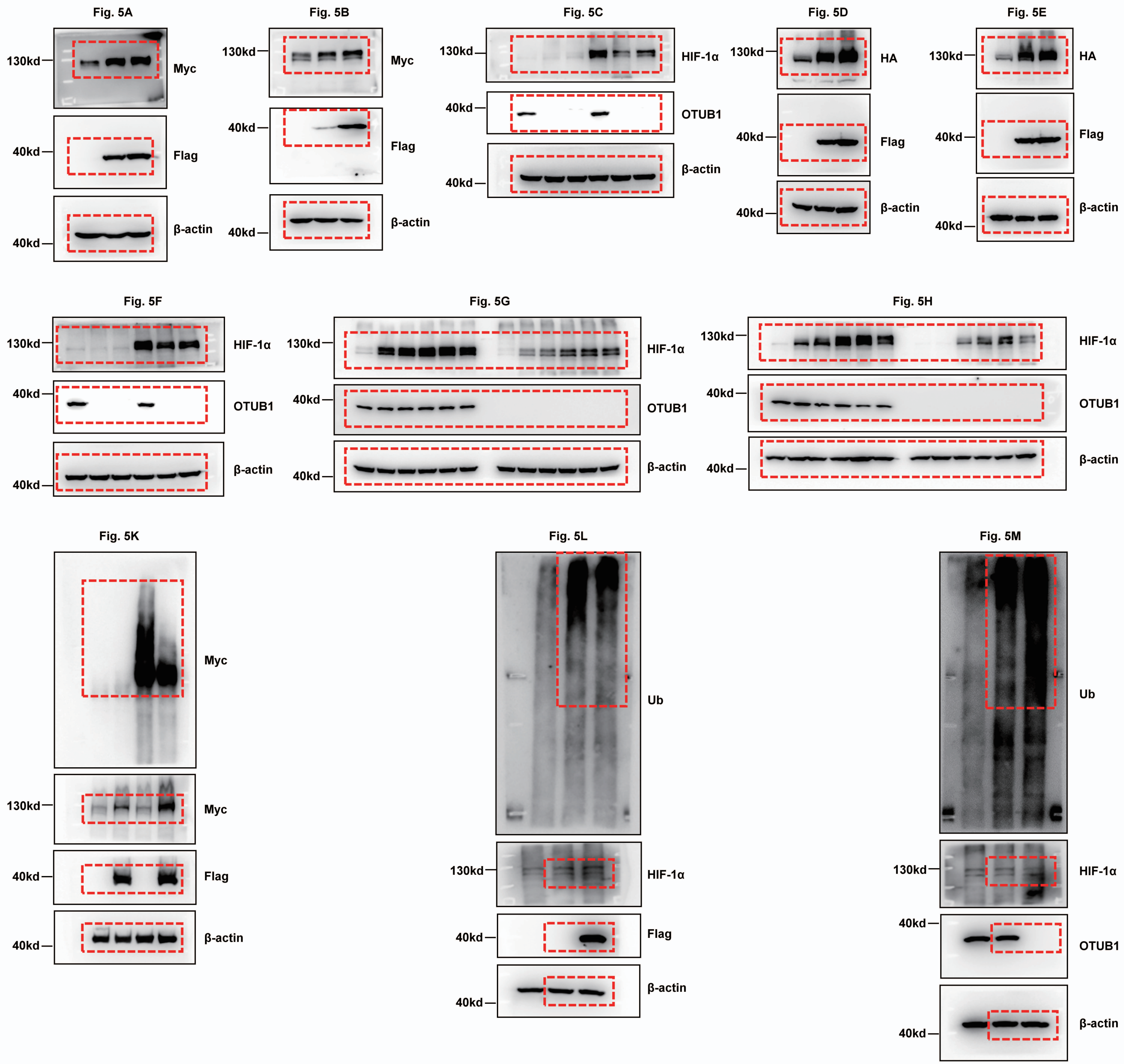

Original Western blot

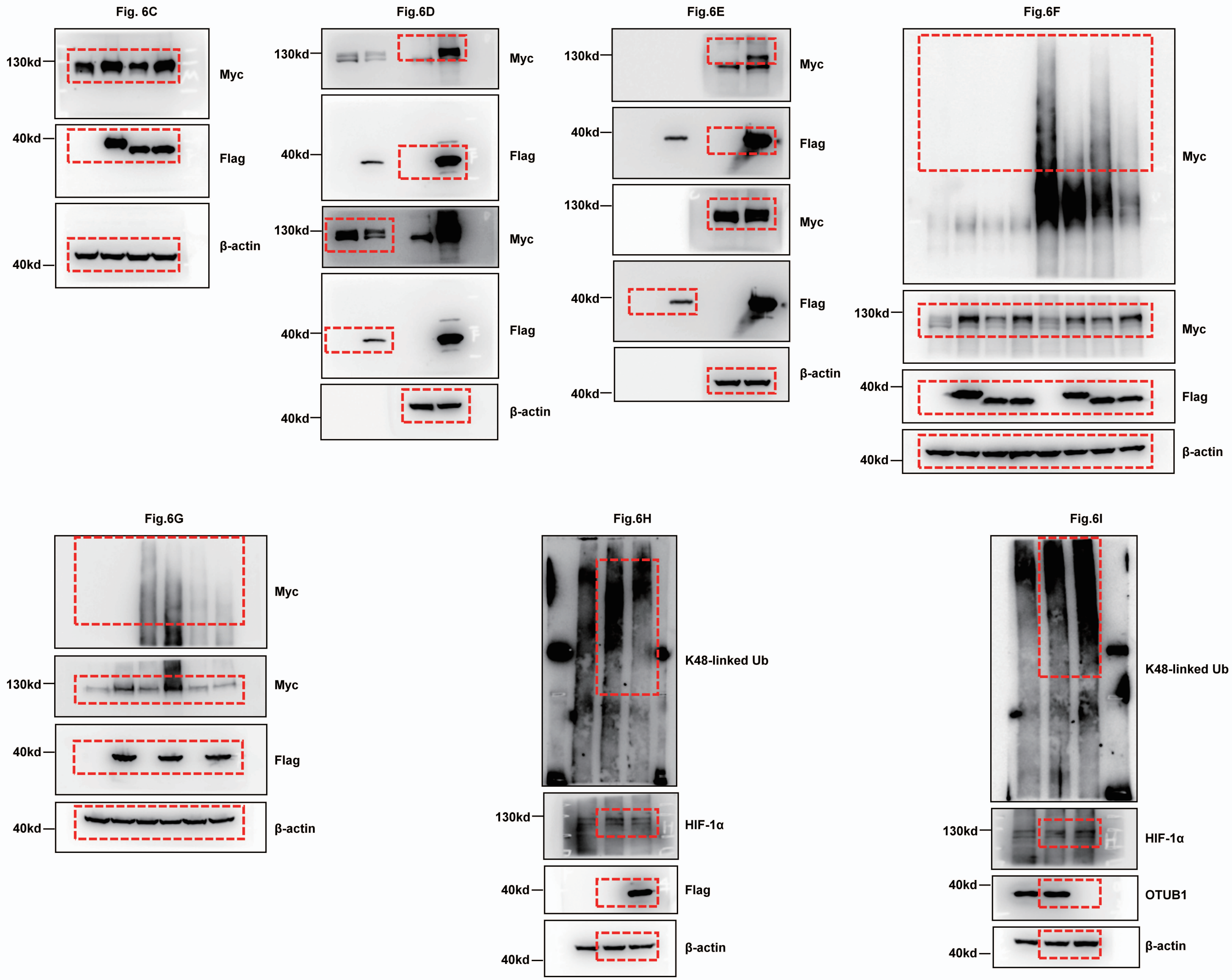

Original Western blot

Fig. S1A

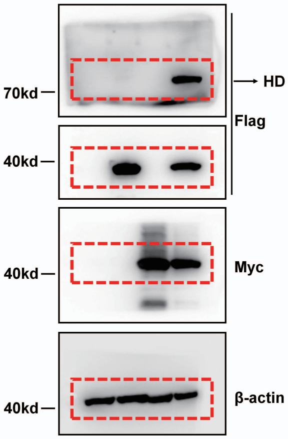

Fig. S1B

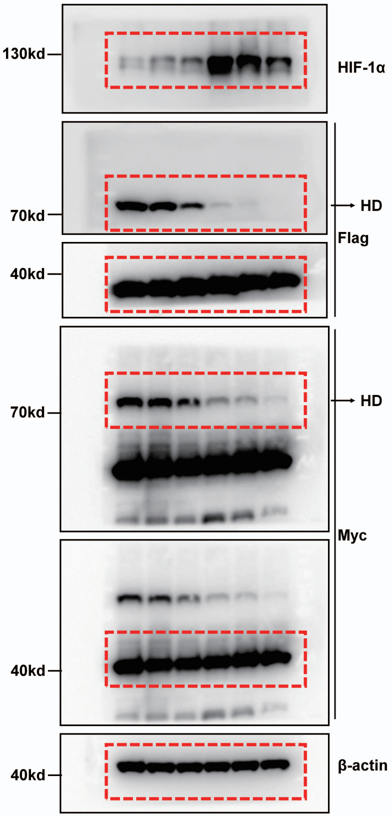

Fig. S1C

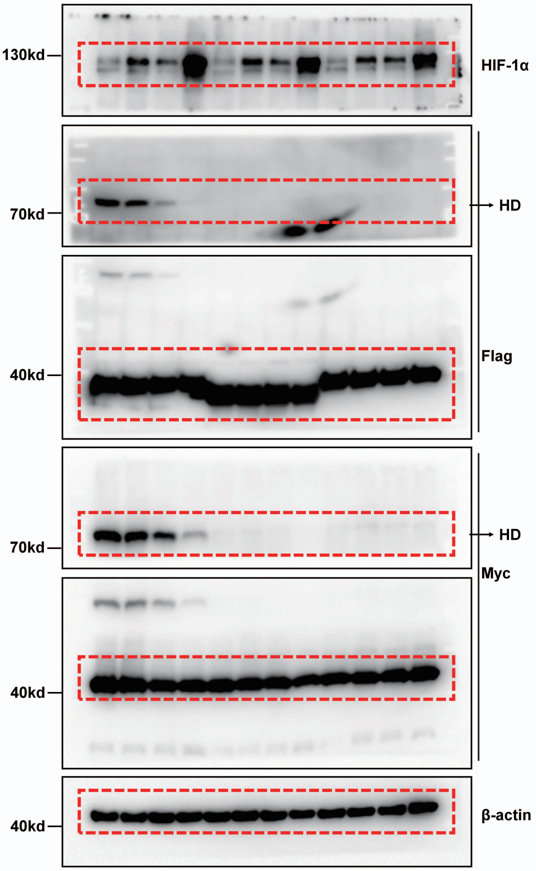

Fig. S2B

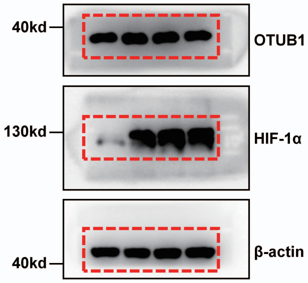

Fig. S3E

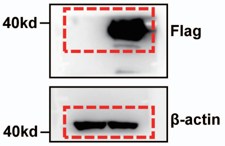

Fig. S3F

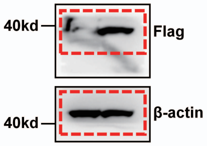

Fig. S4E

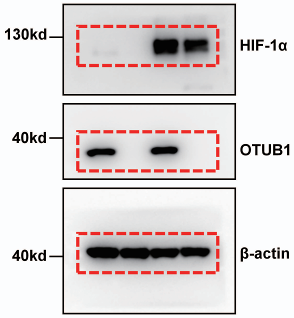

Fig. S4F

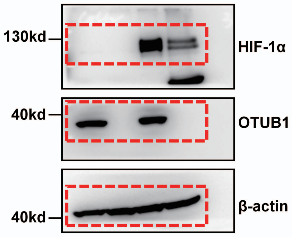

Fig. S4G

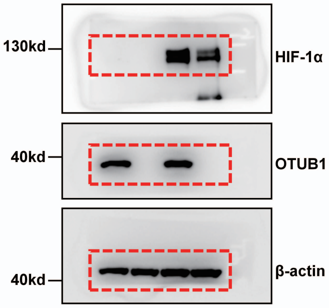

Fig. S4H

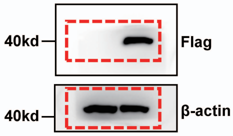

Fig. S4I

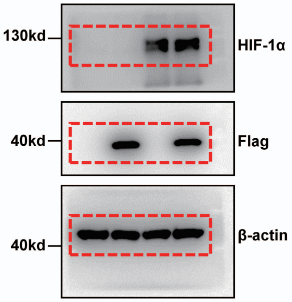

Original Western blot

Fig. S6A

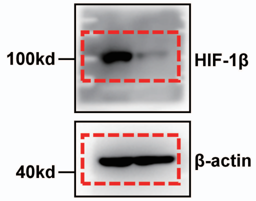

Fig. S6B

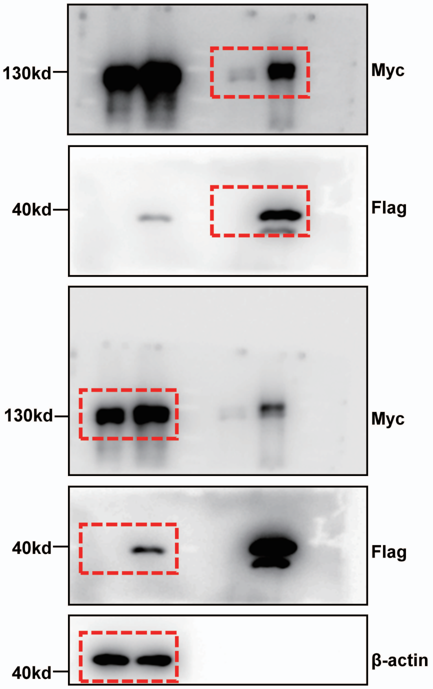

Fig. S6C

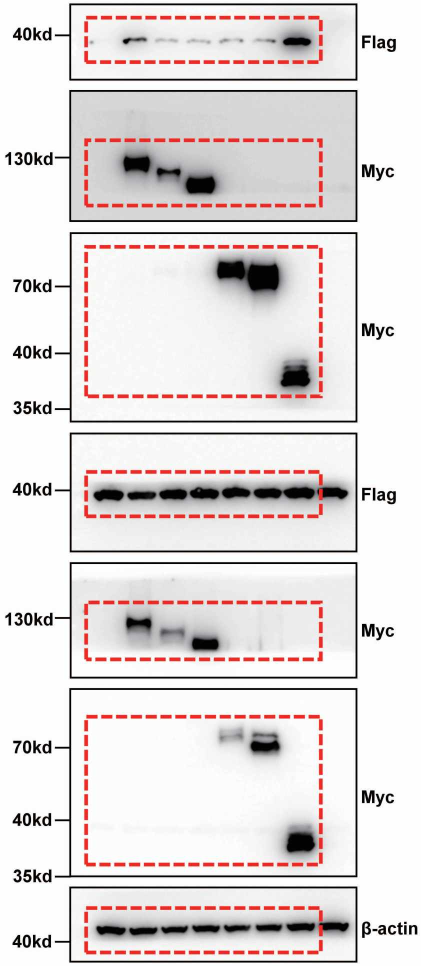

Fig. S7E

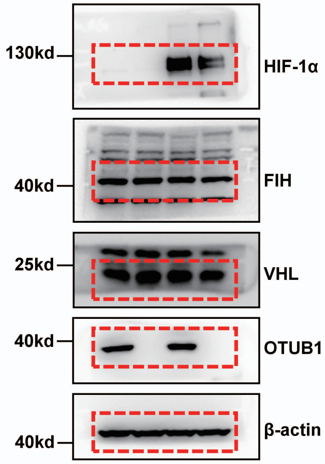

Fig. S7F

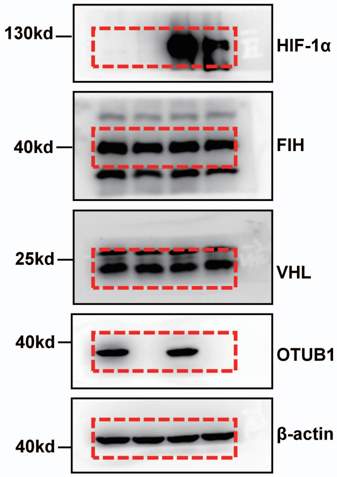

Fig. S7G

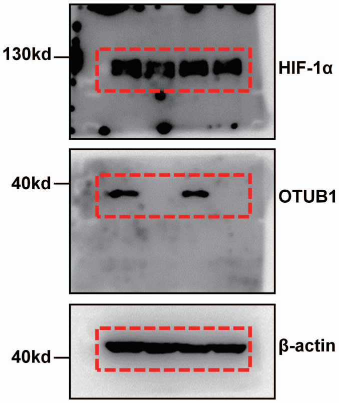

Fig. S9A

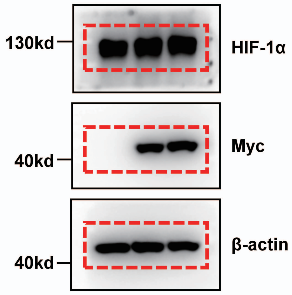

Fig. S9B

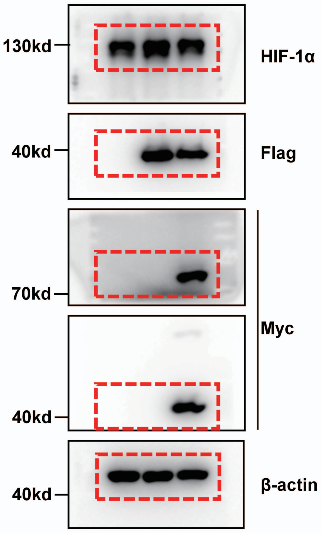

Supplement: Supplementary file 11 — Original Western Blots [file 41419_2022_5008_MOESM11_ESM.pdf]
